# A basic domain in the histone H2B N-terminal tail is important for nucleosome assembly by FACT

**DOI:** 10.1093/nar/gkw588

**Published:** 2016-07-01

**Authors:** Peng Mao, McKenna N. M. Kyriss, Amelia J. Hodges, Mingrui Duan, Robert T. Morris, Mark D. Lavine, Traci B. Topping, Lisa M. Gloss, John J. Wyrick

**Affiliations:** School of Molecular Biosciences and Center for Reproductive Biology, Washington State University, Pullman, WA 99164, USA

## Abstract

Nucleosome assembly *in vivo* requires assembly factors, such as histone chaperones, to bind to histones and mediate their deposition onto DNA. In yeast, the essential histone chaperone FACT (FAcilitates Chromatin Transcription) functions in nucleosome assembly and H2A–H2B deposition during transcription elongation and DNA replication. Recent studies have identified candidate histone residues that mediate FACT binding to histones, but it is not known which histone residues are important for FACT to deposit histones onto DNA during nucleosome assembly. In this study, we report that the histone H2B repression (HBR) domain within the H2B N-terminal tail is important for histone deposition by FACT. Deletion of the HBR domain causes significant defects in histone occupancy in the yeast genome, particularly at HBR-repressed genes, and a pronounced increase in H2A–H2B dimers that remain bound to FACT *in vivo*. Moreover, the HBR domain is required for purified FACT to efficiently assemble recombinant nucleosomes *in vitro*. We propose that the interaction between the highly basic HBR domain and DNA plays an important role in stabilizing the nascent nucleosome during the process of histone H2A–H2B deposition by FACT.

## INTRODUCTION

The fundamental subunit of chromatin is the nucleosome core particle (NCP), in which two copies each of histones H2A, H2B, H3 and H4 are assembled onto 147 bp of DNA. While purified histones and DNA can be efficiently assembled into nucleosomes *in vitro* by salt dialysis, nucleosome assembly at physiological salt concentrations is stimulated by the activity of histone chaperone proteins (1,2). Histone chaperones sequester free histones to prevent nonspecific histone-DNA interactions that can impede nucleosome formation (e.g. (3–5)). Histone chaperones also protect histones from degradation (6) and facilitate their transport to the nucleus (7). During *de novo* nucleosome assembly, chaperone-bound histones are deposited onto genomic DNA in a stepwise pathway: the (H3–H4)_2_ tetramer is deposited first, then two H2A–H2B heterodimers are assembled (2). In addition to delivering histones for assembly, histone chaperones may directly stimulate nucleosome formation by binding to and stabilizing nucleosome assembly intermediates.

The histone chaperone FACT (FAcilitates Chromatin Transcription) is an essential regulator of both nucleosome assembly and disassembly during transcription elongation and DNA replication (8,9). FACT is a heterodimer of the proteins Spt16 and Pob3 in yeast or Spt16 and SSRP1 in humans (9,10). Both Spt16 and Pob3/SSRP1 subunits are highly conserved among eukaryotes and essential for cell viability. Purified FACT can both assemble and disassemble nucleosomes *in vitro* (11,12), and has been shown to induce an alternative nucleosome structure in which the nucleosomal DNA is significantly more accessible (13). While FACT can bind to both H2A–H2B and H3–H4 *in vitro*, it shows a clear preference for H2A–H2B (10,14). Recent structural and biochemical studies have provided conflicting evidence about which residues in the H2A–H2B dimer are important for FACT binding (14–17). FACT must also relinquish its bound histone H2A–H2B to assemble nucleosomes. However, it is not known which histone residues are required for FACT to deposit H2A–H2B during nucleosome assembly.

Previously, we identified a highly basic domain in histone H2B that has important roles in gene expression and DNA repair (18,19). This domain, which is comprised of residues 30–37 in yeast H2B, was named the histone H2B repression (HBR) domain because it functions to repress the expression of nearly 10% of the yeast genome (18,20). The HBR domain is the most conserved region of the H2B N-terminal tail, and is located at a critical juncture between the two DNA gyres of the NCP, where lysine and arginine residues in the HBR domain make numerous contacts with the DNA backbone (20,21). However, histone H2B lacking the HBR domain can be readily assembled into nucleosomes using the artificial 601 nucleosome positioning sequence (22), suggesting that the HBRΔ mutant does not dramatically perturb nucleosome structure. Hence, it is not known how the HBR domain represses the transcription of a large subset of the yeast genome.

Deletion of the HBR domain causes a number of mutant phenotypes in yeast (e.g. hydroxyurea sensitivity, cryptic transcription) that are shared by certain FACT mutants (22). Moreover, a recent study showed that the HBR domain is important for transcription-associated nucleosome disassembly at the yeast *GAL1* promoter and coding region, and is required for FACT to efficiently evict H2A–H2B dimers from nucleosomes *in vitro* (22). Since the HBR deletion had only a marginal effect on the binding affinity of FACT to the H2A–H2B dimer *in vitro* (22), it is unclear how the HBR domain affects FACT activity. Furthermore, it is not known if the HBR domain is required for FACT to assemble nucleosomes, nor if this is a possible mechanism by which HBR represses transcription.

To gain mechanistic insights into the role of the HBR domain in transcriptional repression, we investigated the impact of HBR on nucleosome assembly. ChIP-chip analysis revealed that the HBRΔ mutant caused significant decreases in histone H2B occupancy at HBR-repressed genes and a concomitant increase in H2A–H2B dimers that remain bound to FACT *in vivo*, both of which are consistent with a defect in FACT nucleosome assembly. Furthermore, we discovered that assembly of nucleosomes by FACT on the physiologically relevant 5S rDNA sequence is dependent upon an intact HBR domain. These data suggest that the HBR domain is required for efficient deposition of H2A–H2B dimers onto DNA, which can explain its importance in establishing repressive chromatin *in vivo*.

## MATERIALS AND METHODS

### Yeast strains and plasmids

Wild-type, HBR deletion (*htb1-Δ30-37*), and HBR substitution mutants were constructed by site-directed mutagenesis of plasmids pMP002 (18) and pJW500 (23) using the QuikChange II kit (Agilent) or a modified version of this protocol. Mutations were verified by DNA sequencing. HBRΔ mutant plasmids were introduced into yeast strains by plasmid shuffling (24). Spt16-9xmyc and Nap1-9xmyc tagged strains were constructed by PCR amplification of the 9xmyc-*TRP1* tagging construct from plasmid RY7445 and transformation into yeast strain WY499. Yeast strains and plasmids used in this study are listed in Supplementary Table S1. Primer sequences are available upon request.

### ChIP

Sample preparation for ChIP-chip and ChIP-PCR experiments was performed essentially the same as previously described (25–27), with minor modifications. Briefly, 60 ml of cells were grown in YPD medium to mid-log phase and cross-linked with 1% formaldehyde at room temperature for 15–20 min. Cells were pelleted, washed, snap-frozen in liquid nitrogen and stored at −80°C. To prepare whole cell extracts (WCEs), cell pellets were lysed using glass beads for 2 h at 4°C using a multi-tube vortexer. WCE samples were sonicated to produce DNA fragments an average of 400 bp in size. Chromatin was immunoprecipitated using histone H2B polyclonal antibody (Active Motif, #39237) or histone H3 polyclonal antibody (Abcam, #ab46765) bound to magnetic beads (Dynabeads M-280 Sheep anti-Rabbit IgG, Invitrogen). Following washes and DNA elution, the samples were incubated overnight at 65°C to reverse the formaldehyde crosslinks. The DNA samples were treated with RNase A (Qiagen) followed by Proteinase K (Invitrogen), purified via phenol chloroform extraction and ethanol precipitation. For semi-quantitative PCR, the immunoprecipitated (IP) and WCE control DNA samples were PCR amplified with region-specific primers and control primers that amplified a region of the *ACT1* locus. PCR products were analyzed by agarose gel electrophoresis and quantified using a GelDoc imager (BioRad) and Quantity One software, as previously described (27). While seven regions were initially analyzed, one locus (*SGA1*) was excluded because it showed very faint ChIP-PCR signal.

Chromatin immunoprecipitation quantitative PCR (ChIP-qPCR) were performed similarly, except formaldehyde crosslinking was quenched with 0.125 M glycine, and chromatin was sonicated using the Bioruptor (Diagenode) to generate DNA fragments averaging ∼500 bp. WCEs were immunoprecipitated with an anti-FLAG antibody (Sigma) bound to magnetic beads. The purified IP and WCE samples were analyzed using the ABI 7500 Fast Real-Time PCR system and EvaGreen (Biotium) qPCR mastermix. Region-specific primer sequences are available upon request. Data are presented as percent IP as compared to input, representing at least three replicates.

### Sample preparation for Tiling array analysis

Following histone H2B ChIP, 200 ng of IP or WCE DNA was end repaired using T4 DNA Polymerase (New England Biolabs), and purified by phenol chloroform extraction and ethanol precipitation. Pre-annealed double-stranded linkers OJW102 and OJW103 were ligated to the DNA samples using T4 DNA Ligase (New England Biolabs), and the ligated DNA was purified and concentrated by ethanol precipitation. The samples were amplified by ligation-mediated PCR (LM-PCR) using primer OJW102, a dNTP mix containing dUTP, and AmpliTaq Polymerase (Applied Biosystems). LM-PCR products were purified and concentrated by ethanol precipitation. For each sample, 12 μg of LM-PCR product was fragmented and labeled using the GeneChip WT Double-Stranded DNA Terminal Labeling Kit (Affymetrix) following the manufacturer's instructions. Two IP replicate samples (per strain) and one WCE (i.e. input) sample were hybridized to Affymetrix *Saccharomyces cerevisiae* Tiling 1.0R arrays and scanned.

### Tiling array data normalization and analysis

Tiling array data from duplicate biological samples of wild type and HBR deletion were analyzed using the Affymetrix Tiling Analysis Software (TAS, version 1.1). Probe intensity data for each sample was quantile normalized within (but not between) groups using the TAS software with default settings, except for the following changes: (i) the target intensities were scaled to 500, and (ii) the bandwidth was set at 60. Normalized ChIP-chip data was visualized using the Integrative Genomics Viewer (IGV; (28)). One of the HBR deletion replicates had a decreased/compressed signal distribution relative to the other samples (data not shown), so this replicate was quantile normalized ‘between groups’ with the matched input sample using the TAS software.

Analysis of ‘gene average’ histone occupancy was performed by dividing the coding region of each gene into six equal-sized bins. Two additional bins were included for the promoter (−500 to −251 bp and −250 to −1 bp upstream of transcription start) and two bins for the downstream region (+1 to +250 bp and +251 to +500 bp downstream of transcription termination site). Gene transcription start and termination sites were based on a published data set (29), and were lifted over to the SacCer1 genome annotation (from SacCer2). Microarray probes were assigned to bins associated with each gene, and the ChIP-chip histone occupancy data (log_2_(IP/input)) for each probe was averaged for each bin. The bin values were averaged for each group of yeast genes (i.e., HBR-repressed genes or the rest of the genome). The 521 HBR-repressed genes were derived from our previous study (18). Analysis of histone occupancy near early and late replication origins was performed similarly. Replication origin coordinates were from a published data set (30).

### Co-immunoprecipitation (Co-IP)

Histone chaperone Spt16 or Nap1 was myc-tagged. For each Co-IP assay, ∼50 ml of yeast cells were collected. The cell pellet was resuspended in 600 μl cold Co-IP lysis buffer (40 mM HEPES pH 7.5, 0.1% Tween-20, 150 mM NaCl, 10% glycerol, 1× protease inhibitor cocktail) and WCEs were isolated by vortexing with glass beads. Cell lysates were centrifuged and the soluble supernatant was kept for Co-IP. Protein concentration in the cleared WCEs was measured with the Bradford assay. For Co-IP, ∼3 mg of total proteins were incubated with 30 μl of anti-myc agarose beads (Thermo Scientific) at 4°C overnight. Beads were then washed four times with lysis buffer. Proteins bound on the beads were eluted with 40 ul of 0.1 M glycine (pH 2.4). Eluates were neutralized by adding 5 μl of 1 M Tris–Cl (pH 9.5) and subjected to western blotting.

To analyze the interaction between H2A–H2B dimers and FACT *in vitro*, yeast Spt16 was first immobilized to anti-myc agarose beads as described above. Endogenous yeast histones were stripped with Co-IP lysis buffer containing 500 mM NaCl. Recombinant *Xenopus laevis* H2A and H2B proteins were expressed in *Escherichia coli* cells. Histone expression, purification, and dimer reconstitution were performed as described in a previous report (31). For binding, ∼8 pmol of histone dimers (wild-type or HBR) were incubated with Spt16-bound beads in 400 μl of Co-IP lysis buffer at 30°C for 1 h. After incubation, beads were washed with lysis buffer three times, and proteins were eluted by boiling beads in 1× SDS-PAGE loading buffer.

### Purification of yeast FACT

FACT was purified from an Spt16-TAP (Tandem Affinity Purification) yeast strain using the TAP purification procedure (32). For each purification, 2 L of yeast culture (OD600 of ∼3.0) was spun down and cells were lysed in NP-40 buffer (15 mM Na_2_HPO_4_, 10 mM NaH_2_PO_4_, 1% NP-40, 150 mM NaCl, 2 mM EDTA, 1 mM DTT, 1× Protease inhibitor cocktail) by bead beating. After centrifugation, the cleared cell lysate was incubated with IgG Sepharose fast flow beads (GE Healthcare). FACT bound to the beads was released by digestion with Tobacco Etch Virus protease (TEV protease, Invitrogen) overnight at 4°C. FACT was subsequently bound by Calmodulin Sepharose beads (GE Healthcare) in the presence of 3 mM CaCl_2_. After extensive washes, FACT was eluted with the elution buffer containing 20 mM EGTA. The presence of FACT in each eluted fraction was checked by western blotting with an anti-TAP antibody (Open Biosystems). The peak fractions were combined and FACT was concentrated using Amicon Ultra-0.5 ml concentration columns (Millipore). EGTA was diluted during the concentration process.

### *In vitro* nucleosome assembly with purified FACT

Recombinant *Xenopus laevis* core histone expression, purification, and histone octamer formation was conducted as described previously (33). The 208 bp Lytechinus variegates 5S rDNA was derived from the plasmid pSL208-12 (34), which contains 12 tandem repeats of 5S rDNA and each repeat was separated by an EcoRI digestion site. The 208 bp 5S DNA was labeled with ^32^P at the 5′-end of each strand, using T4 Polynucleotide Kinase (Invitrogen) and [γ-^32^P]ATP (Perkin Elmer). For nucleosome assembly, ∼0.15 pmol of histone octamers (wild-type or HBRΔ) were mixed with FACT (ranging from 62 to 250 ng) in Assembly Buffer (10 mM Hepes pH 7.6, 50 mM KCl, 5 mM MgCl_2_, 0.5 mM EGTA, 0.1 mM EDTA, 10% glycerol, 0.1 mg/ml BSA). Radiolabeled 5S DNA (50 ng) was then added and incubated at 30°C for 75 min. The assembled nucleosome was separated from free DNA by 5% native polyacrylamide gel electrophoresis, and signal was detected using a phosphorimager (Typhoon FLA 7000, GE Healthcare). For oligonucleosome assembly, the 5S-12 DNA released from the plasmid pSL208-12 with BamHI and HindIII digestion was used as the DNA substrate. Radiolabeled 5S-12 (150 ng) was incubated with 0.3 pmol of histone octamers in the presence of FACT. After oligonucleosome assembly incubation, 0.5 μl of EcoRI (10 U/μl, Fermentas) was added and incubated for additional 1 h before electrophoresis.

## RESULTS

### HBR domain affects histone occupancy at HBR-repressed genes and elsewhere in the genome

Our previous study demonstrated that the HBR domain represses the expression of 521 yeast genes, which include many genes involved in vitamin and carbohydrate metabolism or that were adjacent to yeast telomeres (18). Bioinformatics analysis of yeast ChIP-chip data using the ChromatinDB database (35) revealed that HBR-repressed genes tend to have high levels of histone occupancy in their promoter regions (Supplementary Figure S1). We hypothesized that the HBR domain represses transcription by regulating histone occupancy in yeast promoter regions. To test this hypothesis, histone H2B occupancy was profiled by ChIP-chip analysis (36) in wild-type and HBRΔ mutant (H2B Δ30-37) yeast cells.

**Figure 1.**
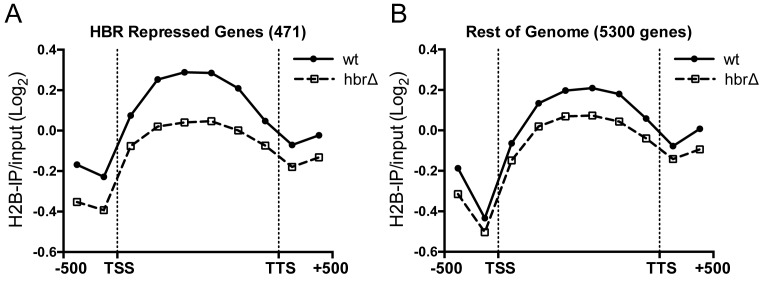
HBR domain affects histone occupancy at HBR-repressed genes and elsewhere in the genome. (**A**) Plot showing average histone H2B occupancy in HBR-repressed genes in wt and HBRΔ mutant. ChIP-chip probe data for HBR-repressed genes (18) with transcript coordinates (471 genes total) were divided into bins representing the promoter region (2 bins), coding region (6 bins), and downstream region (2 bins). ChIP-chip data was averaged for each bin. TSS: transcription start site; TTS; transcription termination site. (**B**) H2B occupancy in genes that are not repressed by HBR domain (5300 genes total).

Wild-type H2B ChIP-chip data (Figure 1) showed the expected trend of lower histone occupancy in promoter regions and higher histone occupancy in the gene body (e.g. (37)). The trend of wild-type H2B occupancy was similar between HBR-repressed genes and genes not repressed by the HBR domain (i.e. ‘Rest of Genome’; compare solid lines in Figure 1A and B). However, at proximal promoter regions (defined as −1 to −250 bp upstream of TSS), wild-type H2B occupancy was ∼15% higher on average for HBR-repressed genes than genes not repressed by the HBR domain (compare solid lines in Figure 1A and B). This finding is in agreement with our analysis of published histone occupancy data (Supplementary Figure S1), which also showed higher histone occupancy in the promoter regions of HBR-repressed genes. There was a similar trend of H2B occupancy in the HBRΔ mutant, but the overall levels of H2B occupancy were reduced relative to wild-type. The decrease in H2B occupancy was particularly apparent in the promoter and coding regions of HBR-repressed genes (Figure 1A). Among yeast genes that are not transcriptionally repressed by the HBR domain (5300 genes), H2B occupancy was only marginally lower in the HBRΔ mutant (Figure 1B). These data are consistent with the hypothesis that the HBR domain is required for high levels of histone occupancy in the promoter and gene body of its target genes to repress their transcription.

To validate the ChIP-chip results, we measured histone H2B occupancy by ChIP-PCR. We analyzed five genomic regions that showed depletion of H2B occupancy in the HBRΔ ChIP-chip experiment (Supplementary Table S2), and one genomic region that was depleted in both wild-type and the HBRΔ mutant (data not shown). As a normalization control for these experiments, we used a region in the *ACT1* ORF in which H2B occupancy was only slightly affected in the HBRΔ mutant. The ChIP-PCR data for these genomic regions confirmed the changes in H2B occupancy in the HBR ChIP-chip experiment (Supplementary Figure S2).

Changes in H2B occupancy could represent a loss of nucleosomes in the HBRΔ mutant or a specific depletion of one or both H2A–H2B dimers from otherwise intact nucleosomes. If the second case were correct, the H3–H4 tetramer should have normal occupancy levels in the HBRΔ mutant. To test this, we measured histone H3 occupancy by ChIP-PCR. Histone H3 occupancy significantly decreased in the HBRΔ mutant at each genomic region tested (Supplementary Figure S2B). Moreover, the magnitude of the change in H3 occupancy largely mirrored the change in H2B occupancy. Taken together, these data suggest that the HBR deletion causes a loss of nucleosome occupancy.

### HBR domain directly regulates histone H2B occupancy

**Figure 2.**
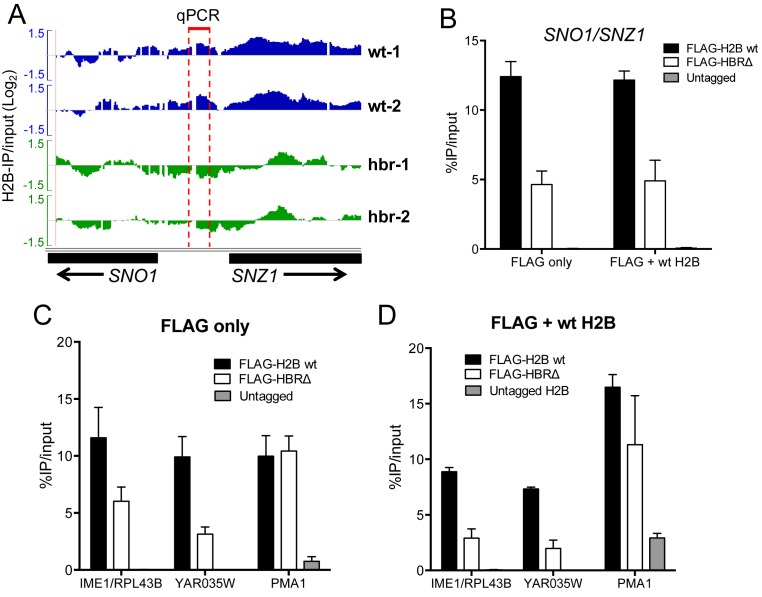
HBR domain directly regulates histone H2B occupancy. (**A**) Snapshot of ChIP-chip data showing decrease in H2B occupancy in the HBRΔ mutant at the bidirectional promoter of HBR-repressed *SNO1* and *SNZ1* genes. Wt-1 and wt-2 and hbr-1 and hbr-2 represent independent ChIP-chip IP data sets from wild-type and HBRΔ mutant cells, respectively. Image was generated using IGV (28). (**B**) ChIP-qPCR confirmation of H2B occupancy defect in HBRΔ mutant at the *SNO1/SNZ1* promoter shown in **A**. ‘FLAG only’ indicates the only source for H2B protein in cells is FLAG-tagged wild-type H2B or HBRΔ mutant. ‘FLAG+wt H2B’ indicates a second plasmid expressing untagged wild-type H2B protein is present in addition to the FLAG-tagged H2B (wild-type or HBRΔ mutant). (**C**) ChIP-qPCR analysis of FLAG-H2B occupancy at three different chromatin loci. ‘FLAG only’ indicates the only source for H2B protein in cells is FLAG-tagged wild-type H2B or HBRΔ mutant. In the ChIP-chip data, H2B occupancy in *IME1/RPL43B* and *YAR035W* is decreased in the HBRΔ mutant, while H2B occupancy at *PMA1* is not affected. These findings were confirmed by ChIP-qPCR. (**D**) Same as **C**, except ‘FLAG + H2B wt’ indicates a second plasmid expressing untagged wild-type H2B is present in all the yeast strains.

Changes in H2B occupancy in the HBRΔ mutant could be a direct effect of the HBR deletion or a secondary consequence of the HBRΔ mutant phenotypes. The HBR deletion strain is slow growing and has significant changes in gene expression (18), which could indirectly influence histone occupancy. To investigate these alternative hypotheses, we analyzed histone occupancy at the bidirectional promoter for the HBR-repressed *SNO1* and *SNZ1* genes. Both the *SNO1* and *SNZ1* transcripts are induced by the HBRΔ mutant (4.7-fold and 4-fold increases, respectively; (18)), and ChIP data confirmed that histone occupancy is decreased at the *SNO1/SNZ1* promoter in the HBRΔ mutant (Figure 2A–B, Supplementary Figure S2B).

To eliminate the possible effects of HBR phenotypes on histone occupancy, we introduced a FLAG-tagged HBRΔ mutant gene into a strain also expressing untagged wild-type histone H2B (Supplementary Figure S3). Expression of wild-type histone H2B eliminated the HBRΔ mutant phenotypes: the heterozygous strain grew normally (Supplementary Figure S4) and was no longer UV sensitive (data not shown). Moreover, quantitative RT-PCR confirmed that HBR-repressed genes were expressed similarly to wild-type in the heterozygous strain (Supplementary Figure S5). ChIP using an anti-FLAG antibody was used to specifically measure occupancy of the HBRΔ mutant histone in this wild-type strain. For comparison, we expressed FLAG-tagged wild-type H2B in a strain also expressing untagged H2B. If the changes in histone occupancy in the HBRΔ mutant were a secondary consequence of HBR phenotypes (e.g. changes in transcription), co-expression of the untagged wild-type H2B should rescue defects in HBR occupancy. However, FLAG-HBRΔ occupancy showed the same decrease at the *SNO1/SNZ1* promoter in the wild-type strain (FLAG + wt H2B) as in the HBRΔ mutant (FLAG only) strain (Figure 2B), indicating that the changes in histone occupancy were a direct effect of the HBRΔ mutant (Supplementary Figure S3B).

To further test this hypothesis, we examined two other genomic regions (*IME1/RPL43B* promoter and *YAR035W* coding region*)* in which H2B occupancy was depleted in the HBRΔ mutant. Again, we observed that FLAG-HBRΔ occupancy is similarly decreased in the wild-type strain (FLAG + wt H2B, Figure 2D) as the HBRΔ mutant strain (FLAG only, Figure 2C). As a control, we measured FLAG-HBR occupancy at a region in the *PMA1* coding region in which H2B occupancy is not depleted in the HBRΔ mutant. ChIP-qPCR data confirmed that the HBRΔ mutant does not affect H2B occupancy at this region in the *PMA1* coding sequence (Figure 2C and D), either in the FLAG only or wild-type strains (*t*-test, *P* > 0.05). In summary, these results indicate that the HBR domain directly regulates histone occupancy at HBR-repressed genes and elsewhere in the genome.

### HBRΔ mutant enhances H2A–H2B binding to FACT, but abolishes Nap1 binding *in vivo*

Loss of histone occupancy in the HBRΔ mutant likely reflects a defect in either nucleosome assembly or stability. Because it is known that histones lacking the HBR domain can form stable nucleosomes with the 601 positioning sequence *in vitro* (22), we hypothesized that the HBRΔ mutant affected histone chaperone binding and assembly activity *in vivo*. Histone chaperones play essential roles in nucleosome assembly during transcription, DNA replication and repair (reviewed in (38)), and thus could be responsible for the defects in histone occupancy observed in the HBRΔ mutant. We focused on the two major H2A–H2B chaperones: FACT and Nap1, and analyzed their interactions with histones *in vivo* using a co-immunoprecipitation (Co-IP) assay. To pull-down Spt16 (a FACT subunit) and Nap1, the chaperones were Myc-tagged and immunoprecipitated with anti-Myc antibody conjugated to agarose beads. Histone binding was detected by western blotting with H2A, H2B or H2AZ-specific antibodies.

**Figure 3.**
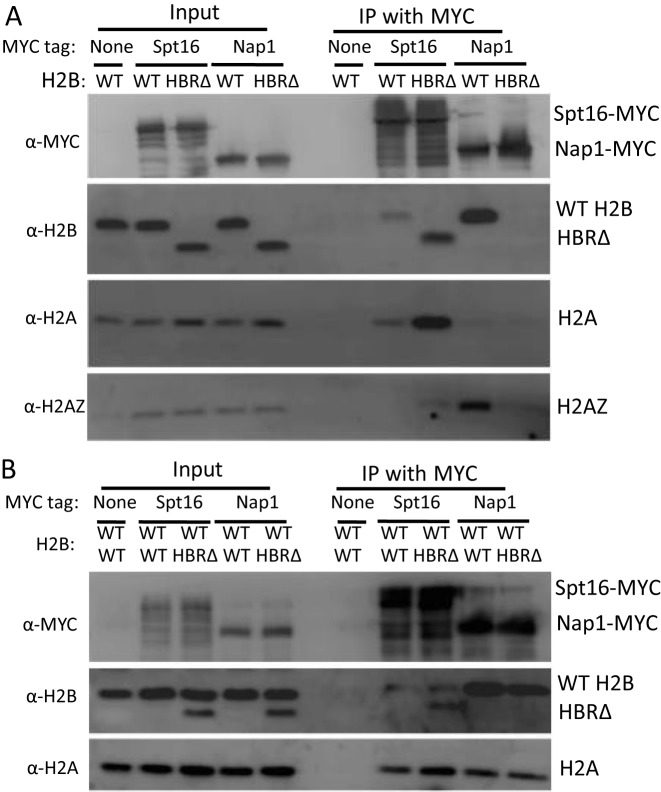
Co-Immunoprecipitation (Co-IP) of wild-type or HBRΔ mutant histones bound to the histone chaperones FACT (Spt16) or Nap1. (**A**) Whole cell extracts from Myc-tagged Spt16 or Nap1 strains were immunoprecipitated (IP) with anti-Myc agarose beads (Thermo Scientific). The abundance of co-immunoprecipitated histones was detected by western blotting using specific antibodies against H2B (Abcam), H2A (Active Motif), and H2AZ (Active Motif). A yeast strain lacking the Myc-tag was used as a negative control. H2B lacking the HBR domain (HBRΔ) migrates faster than the wild-type (WT) H2B protein on the15% SDS gel. (**B**) Same as **A**, except two H2B expression plasmids (either both expressing wild-type H2B or one expressing wild-type and one expressing HBRΔ) were co-expressed in each yeast strain.

Western blot analysis of Spt16 Co-IP samples showed that in wild-type cells, FACT binds both histones H2A and H2B, but not H2AZ (Figure 3A). These results are consistent with previous reports that FACT cannot incorporate H2AZ–H2B dimers into chromatin (39). Neither FACT nor Nap1 bound histone H3 in wild-type cells (Supplementary Figure S6), confirming that they are primarily H2A–H2B (or H2AZ–H2B) chaperones *in vivo*. Interestingly, the binding of FACT to H2A–H2B was increased in HBRΔ mutant cells (Figure 3A). Nap1 preferentially bound to H2AZ–H2B over canonical H2A–H2B in wild-type cells. Strikingly, the binding of Nap1 to H2AZ–H2B (and H2A–H2B, data not shown) was largely abolished in the absence of HBR domain (Figure 3A), indicating that the HBR domain has opposite effects on binding to these two chaperones *in vivo*.

The FACT-histone interactions detected by Co-IP could reflect binding to free histones or DNA-associated histones *in vivo*. Prior to the Co-IP step, the chromatin fraction of the yeast extract is removed by centrifugation (see Materials and Methods). We found that very little genomic DNA was present in the yeast extract supernatant used for the Co-IP experiments, as the DNA was almost entirely associated with the chromatin pellet (Supplementary Figure S7A). To further test whether FACT was bound to DNA-associated or free histones, we digested DNA in the yeast extract with a nonspecific nuclease prior to the centrifugation step. Nuclease treatment increased histone H2B binding to FACT (Supplementary Figure S7B), presumably by releasing more free histones into the supernatant (e.g. (40)). Taken together, these findings suggest that FACT primarily binds free histones in our Co-IP assays, and that the HBRΔ mutant increases the amount of free histone H2A–H2B bound to FACT.

Changes in chaperone binding could be a secondary consequence of mutant phenotypes in the HBRΔ cells (e.g. changes in transcription). To eliminate the possible effects of HBR phenotypes on chaperone binding, we repeated the Spt16 and Nap1 Co-IP experiments in a strain expressing both wild-type H2B and HBRΔ mutant histones. This presence of wild-type H2B eliminated the HBRΔ mutant phenotypes (Supplementary Figures S4 and S5), and enabled us to directly compare the chaperone binding affinity of the wild-type and HBRΔ mutant histones. In yeast cell extracts (Input), protein bands corresponding to wild-type H2B and HBRΔ mutant can be readily separated by SDS-PAGE and detected by western blotting using an anti-H2B antibody (Figure 3B). HBRΔ mutant protein levels were considerably lower than wild-type H2B (∼40% of wild-type H2B protein level) when co-expressed (Figure 3B). Importantly, the amount of HBRΔ mutant that was bound to FACT was higher than that of wild-type H2B, particularly after normalizing to input (Figure 3B). These data show that deletion of the HBR domain enhances FACT binding *in vivo*. In contrast, Nap1 only bound to wild-type H2B (Figure 3B), confirming that the HBR domain is required for Nap1 binding.

The increase in histone binding to FACT in the HBRΔ mutant could also be caused by the loss of Nap1 binding to the mutant dimer. To test this hypothesis, *NAP1* was deleted in the Spt16-Myc cells, and Co-IP experiments were used to compare the levels of H2B binding to FACT in wild-type and *nap1Δ* cells. The *nap1* deletion did not increase the binding of histone H2A or H2B to FACT (Supplementary Figure S8), suggesting that the increased binding of HBRΔ mutant histones to FACT is not caused by the loss of binding to Nap1.

### HBR deletion does not increase FACT-H2A/H2B binding *in vitro*

**Figure 4.**
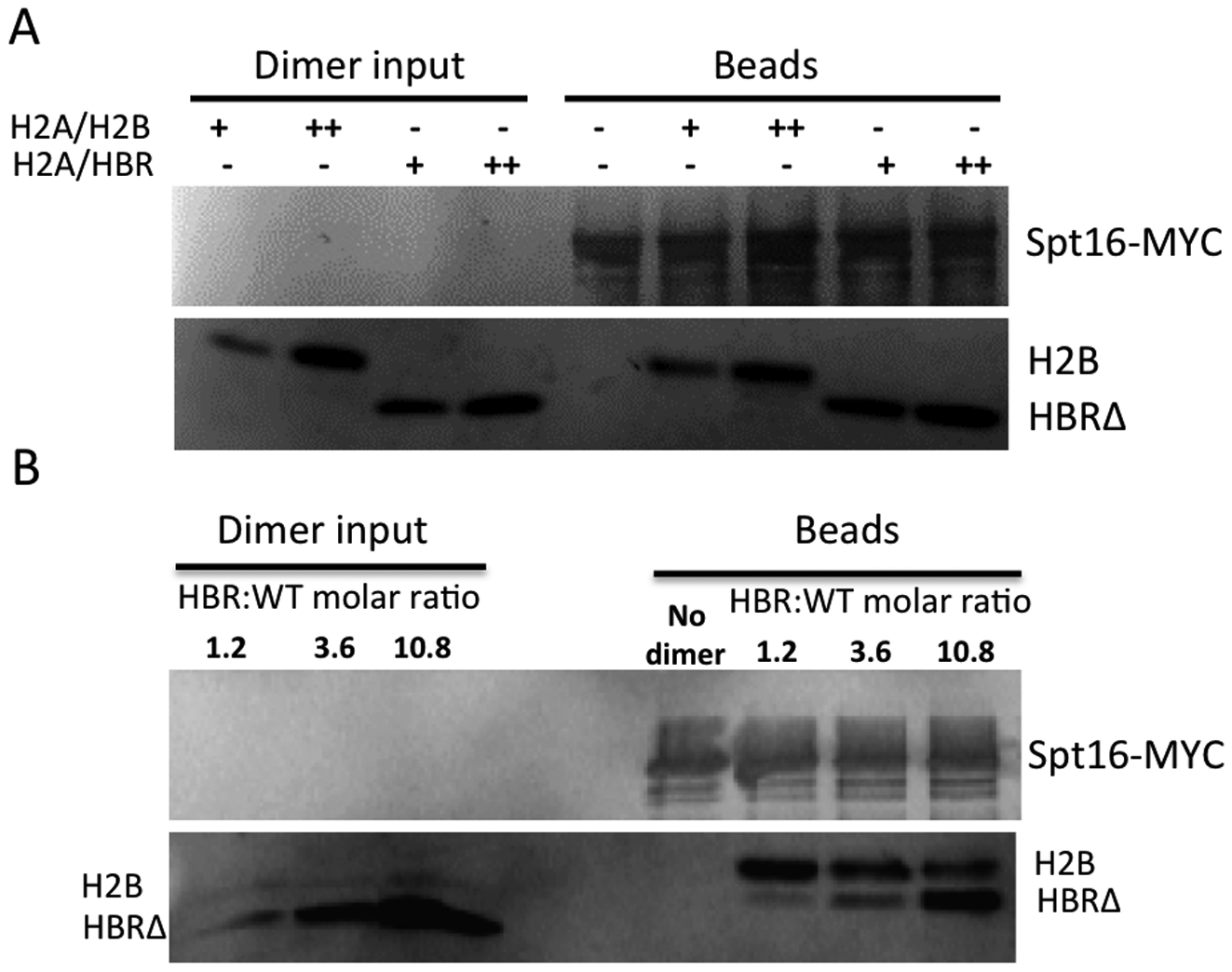
*In vitro* binding of FACT to wild-type H2A/H2B and mutant H2A/HBRΔ dimers. (**A**) Western blots showing the binding of FACT to wild type (H2A/H2B) and HBR-deleted mutant (H2A/HBR) histone dimers. Wild-type or mutant dimers were incubated separately with FACT (Spt16-Myc) bound to anti-Myc agarose beads. Two histone dimer concentrations, 100 nM (+) and 200 nM (++) were used in this assay. Western blot analysis was used to detect FACT-bound histones (‘Beads’). 'Dimer input’ shows the level of wild-type or mutant dimers present in the reaction before incubating with FACT. (**B**) Competition between wild-type and mutant histone dimers for FACT binding. Wild-type and mutant histone dimers were mixed at different molar ratios and subsequently incubated with FACT bound to anti-myc agarose beads. The concentration of wild-type dimer was kept at 100 nM while the mutant dimer concentrations were 120, 360 and 1080 nM, respectively.

The simplest interpretation of our Spt16 Co-IP data is that the HBR domain inhibits FACT binding to the H2A–H2B dimer. To test if HBR directly affects FACT binding to the H2A–H2B dimer, we performed an *in vitro* binding assay using purified FACT and recombinant *Xenopus* histone dimers (Supplementary Figure S9). FACT was purified with anti-Myc beads from yeast extracts, and endogenous yeast histones bound to FACT were removed using a high-salt wash. Equal moles of recombinant wild-type or HBRΔ mutant dimers were added and incubated with FACT immobilized on agarose beads, in order to compare the binding affinity of the HBRΔ mutant dimer to wild-type. Both wild-type and HBRΔ mutant dimer bound similarly to FACT using two different dimer concentrations (Figure 4A), which is consistent with published data (22).

The affinity of wild-type and HBRΔ mutant dimers to FACT was further compared using a binding competition assay, where a mixture of wild-type and HBRΔ mutant dimers were incubated with FACT immobilized on agarose beads. Intriguingly, when the molar ratio of mutant to wild-type dimer (HBR:WT) was 1.2:1 or even 3.6:1, FACT primarily bound wild-type H2B (Figure 4B). Only when the molar ratio was 10.8:1 (HBR:WT) did more HBRΔ mutant bind FACT (Figure 4B). The *in vitro* binding results indicate that the HBR deletion does not directly enhance binding of the H2A–H2B dimer to FACT; rather, the HBRΔ mutant dimer is less competitive for binding FACT in the presence of wild-type dimer.

### HBR domain is important for FACT-mediated nucleosome assembly on 5S rDNA

Because the HBR deletion does not promote binding to FACT *in vitro*, we tested the alternative hypothesis that FACT is unable to efficiently deposit the HBRΔ mutant dimer onto DNA, which could lead to an accumulation of mutant dimer bound to FACT *in vivo*. A previous study has shown that purified human FACT can deposit *Xenopus* histones onto a 5S ribosomal DNA (rDNA) template to assemble nucleosome core particles (NCPs) (12). We first tested if yeast FACT is also able to assemble NCPs using reconstituted *Xenopus* histone octamers and the 208 bp 5S rDNA. FACT was purified from yeast cells using the TAP purification procedure (Supplementary Figure S10A). Incubation of recombinant histone octamer and 5S rDNA in the presence of purified FACT resulted in assembled NCPs that were the same size as the 5S NCPs formed by salt-dialysis (Supplementary Figure S10B). Importantly, no NCP formation was detected when FACT was absent in the reaction.

**Figure 5.**
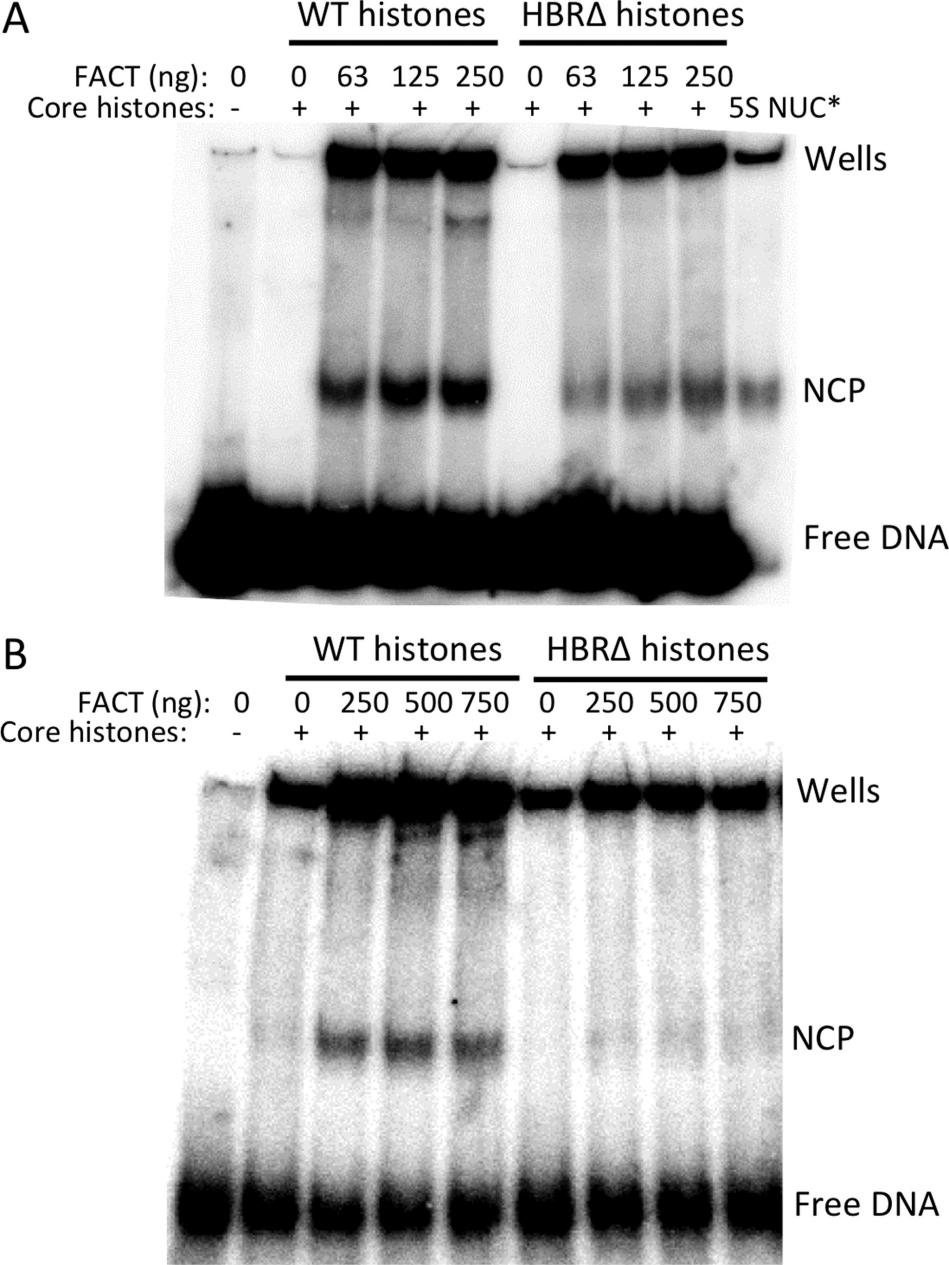
*In vitro* nucleosome assembly using purified yeast FACT complex. (**A**) Mono-nucleosome assembly on radiolabeled 208-bp 5S rDNA. ‘NCP’ indicates the position of nucleosome core particle formed by FACT or salt dialysis. ‘Free DNA’ indicates the position of 5S DNA that was not assembled into nucleosomes. ‘5S NUC*’ indicates NCPs that are formed through standard salt dialysis. (**B**) Oligo-nucleosome assembly on the 5S-12 DNA substrate.

To determine whether the HBR domain was important for FACT to assemble nucleosomes, increasing concentrations of FACT were titrated into nucleosome assembly reactions containing 0.15 pmol of wild-type or HBRΔ mutant histone octamers, and subsequently mixed with 0.3 pmol of 5S rDNA. NCP formation by FACT was clearly less efficient with HBRΔ mutant histones than wild-type histones (Figure 5A). Quantification of three independent experiments indicates that the level of assembled mutant NCPs was only ∼30% of wild-type NCPs for the lowest FACT concentration (Figure 5A, 63 ng FACT). Even at higher FACT concentrations, mutant NCP formation was still significantly lower than wild-type (Figure 5A).

We also examined whether the defect in nucleosome assembly for the HBRΔ mutant histones simply reflected slower kinetics of nucleosome assembly. Time course experiments revealed that nucleosomes were assembled rapidly by FACT (Supplementary Figure S11). Both wild-type and mutant NCPs were detected almost immediately after FACT, histones, and DNA were mixed, indicating that NCP assembly by FACT occurs very rapidly in this assay. Importantly, the formation of HBRΔ mutant NCPs was significantly lower than that of wild-type NCPs through the entire time course (Supplementary Figure S11).

Native gel electrophoresis of the assembled NCPs revealed a super-shifted band that was present in the wild-type samples but not in HBRΔ samples (e.g. Figure 5A and Supplementary Figure S11). This band likely includes FACT, as its intensity increased with higher FACT concentrations (Figure 5A), and was not observed in the absence of FACT (see lanes 2 and 5 in Supplementary Figure S10). It is unlikely to be simply a FACT-DNA complex, as it was not observed in the absence of histones (see lane 3 in Supplementary Figure S10). Therefore, we hypothesize that the super-shifted band is a complex of FACT bound to the assembled NCP. The super-shifted band was not observed when FACT was incubated with pre-assembled NCPs (data not shown), although the FACT accessory factor Nhp6, which is required for FACT to bind intact nucleosomes (41), was present in our purified FACT complex, albeit at sub-stoichiometric levels (Supplementary Figure S10A). It is possible that the super-shifted band represents FACT bound to a partially assembled nucleosome (see Discussion).

We also examined the effect of the HBRΔ mutant on oligonucleosome assembly using a template containing 12 copies of the 5S rDNA sequence (5S-12 DNA) (34). The experimental strategy for oligonucleosome assembly was essentially the same as for mononucleosome, except an Eco*RI* digestion step was required to release mononucleosome sized fragments prior to native gel electrophoresis. Consistent with the mononucleosome data, HBR deletion significantly reduced FACT activity in oligonucleosome assembly (Figure 5B).

### Affinity of DNA for basic residues in HBR domain is important for nucleosome assembly and cell viability

Because HBR is a highly basic domain (six basic amino acids out of the total eight residues) that interacts with both DNA gyres in the nucleosome (18,20), we reasoned that the nucleosome assembly defect could be due to a weakened interaction between the H2A–H2B dimer and the nucleosomal DNA. To test this hypothesis, we investigated whether a higher affinity nucleosome positioning sequence would rescue the HBRΔ mutant assembly defect. We chose the Widom 601 DNA, an artificial DNA sequence that binds to histones with significantly higher affinity than 5S rDNA (42,43). Mononucleosome assembly reactions showed that FACT can assemble both wild-type and HBRΔ mutant histones onto 601 DNA with comparable efficiency (Supplementary Figure S12A), indicating that the strong histone affinity of 601 DNA can compensate for the assembly defect caused by the HBR deletion. Moreover, when NCPs were assembled using the standard salt-dialysis method (44), HBRΔ mutant histones were assembled into NCPs efficiently with 601 DNA, but inefficiently with 5S rDNA (Supplementary Figure S12B), supporting the hypothesis that the HBR domain is required for nucleosome assembly with lower affinity DNA sequences.

We also tested whether basic residues in the HBR domain were critical for HBR function *in vivo*. The eight residues comprising the HBR domain were mutated in tandem to all alanine (nonpolar side chains), all serine (polar side chains), all glycine (no side chain), or all lysine (positively charged side chains; see Table 1). The all-alanine, all-serine, and all-glycine HBR mutants were lethal in yeast (Table 1). However, the all-lysine HBR mutant was viable and grew normally, unlike the slow-growing HBR deletion mutant. The HBR deletion mutant is also hypersensitive to ultraviolet (UV) light and the replication inhibitor hydroxyurea (HU) (18,22). However, the all-lysine HBR mutant showed similar UV and HU sensitivity as wild-type (Supplementary Figure S13). Taken together, these results indicate that the positive charge of the basic residues in the HBR domain is critical for HBR function *in vivo*.

**Table 1.** Yeast viability with different HBR deletions or substitutions

Strain	H2B N-terminal Sequence	Yeast Viability
Wild type H2B	SAKAEKKPASKAPAEKKPAAKKTSTSTDG**KKRSKARK**ETY	**+**
H2B Δ30-37	SAKAEKKPASKAPAEKKPAAKKTSTSTDG--------ETY	**+**
H2B Δ3-37	SA-----------------------------------ETY	**+**
H2B 30–37lys	SAKAEKKPASKAPAEKKPAAKKTSTSTDG**KKKKKKKK**ETY	**+**
H2B 30–37gly	SAKAEKKPASKAPAEKKPAAKKTSTSTDG**GGGGGGGG**ETY	**–**
H2B 30–37ser	SAKAEKKPASKAPAEKKPAAKKTSTSTDG**SSSSSSSS**ETY	**–**
H2B 30–37ala	SAKAEKKPASKAPAEKKPAAKKTSTSTDG**AAAAAAAA**ETY	**–**

## DISCUSSION

**Figure 6.**
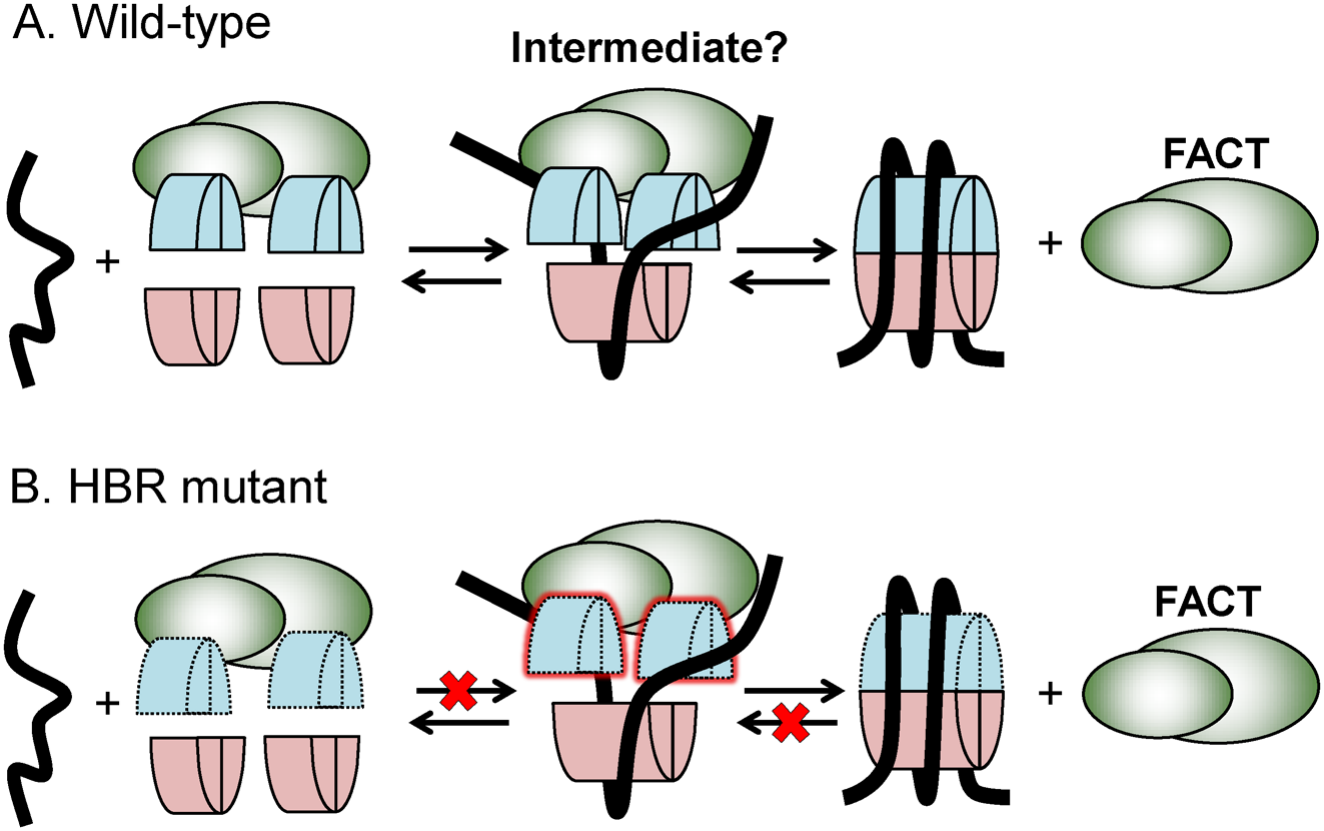
Model of the mechanism by which the HBR domain affects nucleosome assembly and disassembly by FACT. Histone H2A–H2B dimers are in blue; and H3–H4 tetramer is in pink. DNA is represented as a black line, and the FACT heterodimer is in green. In this model, the (**A**) wild-type HBR domain stabilizes a partially assembled nucleosome intermediate that is required for both assembly and disassembly pathways. (**B**) In the HBR mutant (dotted outline of H2A–H2B dimers), the nucleosome intermediate is destabilized, potentially due to weakened histone–DNA interactions, thus inhibiting both nucleosome assembly and disassembly pathways.

Histone proteins are deposited onto DNA by histone chaperones to form nucleosomes, but the molecular mechanism of histone deposition is unclear. In this study, we discovered that the HBR domain in the histone H2B N-terminal tail is important for histone deposition and nucleosome assembly by the essential chaperone FACT. Deletion of the HBR domain causes significant loss of histone occupancy (H2B and H3) at many loci in the yeast genome, particularly genes repressed by HBR. Concomitantly, binding of H2A–H2B dimers to FACT increased *in vivo*. We determined that the HBR domain is required for purified FACT to efficiently assemble recombinant histones into nucleosomes with the 5S rDNA sequence. The defect in FACT assembly of HBR mutant nucleosomes likely reflects a decrease in affinity of HBR mutant histones for nucleosomal DNA, as FACT can efficiently assemble nucleosomes containing HBR mutant histones on a higher affinity DNA sequence (601 template). Because the HBR domain is also important for FACT to disassemble nucleosomes (22), we hypothesize that basic residues in the HBR domain stabilize a critical nucleosome intermediate that is common to both the nucleosome assembly and disassembly pathways (Figure 6).

The HBR domain was previously identified because of its widespread role in repressing yeast transcription (18). Our results indicate that the HBR domain is required for the proper assembly of repressive chromatin at HBR-repressed genes, as deletion of the HBR domain causes a significant loss of histone occupancy, particularly at HBR-repressed promoters. Furthermore, experiments measuring FLAG-HBR occupancy in the presence of untagged wild-type H2B indicate that changes in histone occupancy can be directly attributed to HBR function in nucleosome assembly and/or stability, at least for the genomic regions we tested. Our data also indicate that HBR regulates histone H3 occupancy, as we observed a similar decrease in H3 occupancy in the HBRΔ mutant. Since binding of H3 to the chaperone Asf1 is increased in the HBRΔ mutant cells (22), impaired H2A–H2B deposition in the HBRΔ mutant may cause partially assembled nucleosomes containing H3-H4 to be unstable and eventually disassembled by histone chaperones such as Asf1.

It is not clear why the chromatin structure at HBR-repressed genes is particularly sensitive to perturbation of HBR function. One possibility is that these DNA sequences have lower intrinsic affinity for histones. Alternatively, these genes may require very efficient nucleosome assembly to ensure transcriptional repression. It is notable that the HBR deletion mutant affects histone occupancy at many loci, not just HBR-repressed genes. Indeed, our preliminary analysis indicates that the HBRΔ mutant also affects histone occupancy near yeast replication origins (Supplementary Figure S14), which is consistent with the postulated role of FACT in nucleosome assembly during DNA replication (9).

The HBR domain has recently been shown to be important for FACT to disassemble nucleosomes (22). This study observed an increase in histone occupancy/retention at transcriptionally activated genes in yeast (e.g. *GAL1*) in the HBRΔ mutant and a defect in nucleosome disassembly by FACT *in vitro* (22). Our ChIP-chip data indicate that the HBRΔ mutant primarily causes a loss of histone occupancy in the yeast genome, although histone occupancy is increased at a few loci (data not shown). This suggests that the major function of the HBR domain is in nucleosome assembly, which is consistent with gene expression studies indicating that HBR primarily represses transcription (18). However, HBR's role in nucleosome disassembly is likely to be more prominent during rapid transcriptional activation of specific yeast genes (e.g. *GAL1*).

A critical question is how the same histone domain is important for FACT activity in both nucleosome assembly and disassembly. One possible explanation is that the HBR domain is required for FACT binding. However, our *in vitro* studies indicate that FACT can readily bind HBRΔ mutant histones, albeit more weakly than wild-type histones in competition assays. A previous study, using a quantitative fluorescence dequenching assay, found that the HBRΔ mutant dimer had roughly similar binding affinity to FACT as wild-type dimer (25.4 nM versus 21.4 nM, (22)), which is consistent with our results. Moreover, our *in vivo* data indicate that FACT binds to more HBRΔ mutant dimer than wild-type dimer, possibly because the HBRΔ mutant dimer is not efficiently deposited by FACT onto DNA. This model could explain the different results obtained from competition binding assays *in vivo* (Figure 3B) and *in vitro* (Figure 4B). Alternatively, it is possible that the HBRΔ mutant alters *in vivo* histone post-translational modifications that regulate FACT binding or activity (e.g. (37,45)). Taken together, these data do not support the hypothesis that HBRΔ mutant histones are unable to productively bind to FACT.

Instead, we propose that the HBR domain is important to stabilize a partially assembled nucleosome intermediate that is formed by FACT in both its nucleosome assembly and disassembly pathways (Figure 6). Our data suggest that favorable electrostatic interactions between the basic residues in the HBR domain and the nucleosomal DNA may be critical to stabilizing such an intermediate. It is tempting to speculate that the super-shifted band detected in wild-type nucleosome assembly reactions, but absent in the HBRΔ mutant (see Figure 5 and Supplementary Figures S10 and S11) represents FACT bound to a partially assembled nucleosome intermediate. In any case, these findings demonstrate that the HBR domain in histone H2B is important for FACT to efficiently assemble nucleosomes. The importance of HBR in nucleosome assembly can potentially explain not only its role in transcriptional repression, but also provide new insight into its potential roles in DNA replication and the DNA damage response (18–19,22).

## Supplementary Material

SUPPLEMENTARY DATA

## ACCESSION NUMBER

GEO GSE80831.
