# Supplementary material for: A basic domain in the histone H2B N-terminal tail is important for nucleosome assembly by FACT
Source: Nucleic Acids Res. 2016 Jul 1;44(19):9142–52. doi: 10.1093/nar/gkw588 (PMC5100577; doi:10.1093/nar/gkw588)
Supplement: SUPPLEMENTARY DATA [file supp_44_19_9142__index.html]

A basic domain in the histone H2B N-terminal tail is important for nucleosome assembly by FACT — A basic domain in the histone H2B N-terminal tail is important for nucleosome assembly by FACT — SUPPLEMENTARY DATA 

# A basic domain in the histone H2B N-terminal tail is important for nucleosome assembly by FACT

## SUPPLEMENTARY DATA

- SUPPLEMENTARY DATA
