# Supplementary material for: A basic domain in the histone H2B N-terminal tail is important for nucleosome assembly by FACT
Source: Nucleic Acids Res. 2016 Jul 1;44(19):9142–52. doi: 10.1093/nar/gkw588 (PMC5100577; doi:10.1093/nar/gkw588)
Supplement: SUPPLEMENTARY DATA [file supp_gkw588_nar-01362-m-2016-File008.pdf]

SUPPLEMENTARY MATERIALS AND METHODS

SUPPLEMENTARY METHODS

Quantitative RT-PCR Analysis of HBR-repressed genes. Yeast strains (WT: ALY018, HBRΔ: ALY024, WT+WT: ALY054, WT+HBRΔ: ALY058) were grown to log phase. Cells were collected and washed with sterile diH_2O. RNA was isolated with acid phenol (phenol: chloroform: isoamyl alcohol=125: 24: 1, pH 4.3) at 65 C. RNA was treated with DNase I to remove residual genomic DNA. About 1 ug of RNA was used for reverse transcription with SuperScript III reverse transcriptase (Thermo Fisher). The synthesized cDNA was used for real-time PCR to detect expression of *SNZ1*, *SNO1*, *GCY1*, three HBR-repressed genes revealed in our previous studies, and *ACT1*, an internal loading control. *SNZ1*, *SNO1*, and *GCY1* mRNA levels were normalized to *ACT1*.

Supplementary Table S1. Yeast strains and plasmids.

Strain Name	Genotype	Source
PY013 (WT)	*MATa ade2-1 trp1-1 can1-100 leu2-3,112 his3-11,15 ura3 hta1,htb1Δ::HIS3 hta2,htb2Δ::LEU2 plus pMP011 (CEN6 URA3 HTA2 HTB2)*	(1)
PY014 (WT)	*Isogenic to PY013 plus pMP002 (CEN6 TRP1 HTA1 HTB1)*	(1)
PY020 (H2B Δ30-37 [HBRΔ])	*Isogenic to PY013, plus pMP031 (CEN6 TRP1 HTA1 htb1 Δ30-37)*	(1)
YDL039 (H2B 30-37lys)	*Isogenic to PY013, plus pDL039 (CEN6 TRP1 HTA1 htb1 30-37lys)*	This study
ALY053 (WT)	*MATa ade2-1 trp1-1 can1-100 leu2-3,112 his3-11,15 ura3-1 hta1,htb1Δ::HIS3 hta2,htb2Δ::LEU2 bar1Δ::KanMx4 plus pMP002 (CEN6 TRP1 HTA1 HTB1) and pAL016 (CEN6 ADE2 HTA1 htb1-MYC)*	This study
ALY054(WT + WT H2B FLAG)	*Isogenic to ALY053 but with plasmids pmp002 (CEN6 TRP1 HTA1 HTB1) and pAL017 (CEN6 ADE2 HTA1 FLAG-HTB1)*	This study
ALY058 (WT +H2B Δ30-37 [HBRΔ]-FLAG)	*Isogenic to ALY053 but with plasmids pmp002 (CEN6 TRP1 HTA1 HTB1) and pAL039 (CEN6 ADE2 HTA1 FLAG-htb1Δ30-37)*	This study
ALY016 (WT)	*Isogenic to ALY053 but with plasmid pMK001 (CEN6 ADE2 HTA1 HTB1)*	This study
ALY018 (H2B-FLAG)	*Isogenic to ALY053 but with plasmid pAL017 (CEN6 ADE2 HTA1 FLAG-HTB1)*	This study
ALY024 (H2BΔ30-37[HBRΔ]-FLAG)	*Isogenic to ALY053 but with plasmid pAL039 (CEN6 ADE2 HTA1 FLAG-htb1Δ30-37)*	This study
MP006 (Spt16-MYC + WT H2B)	*MATa his3-1,leu2-0, met15-0, ura3-0, hht1-hhf1Δ::KAN, hhf2-hht2Δ::NAT, hta1-htb1Δ::HPH, hta2-htb2Δ::NAT, TRP1Δ::HIS3, Spt16-9*MYC with histone plasmid pJW500 (CEN6, LEU2, HTA1-HTB1,HHF2-HHT2)*	This study
MP007 (Nap1-MYC + WT H2B)	*Isogenic to MP006 but Nap1 is tagged with 9*MYC*	This study
MP008 (Spt16-MYC + HBRΔ)	*Isogenic to MP006 but with plasmid pTO13 (CEN6, LEU2, HTA1-htb1Δ30-37, HHF2-HHT2)*	This study
MP009 (Nap1-MYC + HBRΔ)	*Isogenic to MP008 but Nap1 is tagged with 9*MYC*	This study
MP010 (Spt16-MYC + two WT H2B)	*Isogenic to MP006 but with one additional plasmid pJH33 (CEN6, URA3, HTA1-HTB1, HHF2-HHT2)*	This study
MP011 (Nap1-MYC + two WT H2B)	*Isogenic to MP007 but with the additional plasmid pJH33*	This study
MP012 (Spt16-MYC + one WT H2B and one HBRΔ)	*Isogenic to MP008 but with the additional plasmid pJH33*	This study
MP013 (Nap1-MYC + one WT H2B and one HBRΔ)	*Isogenic to MP009 but with the additional plasmid pJH33*	This study

MP026 (Spt16-MYC in WT Nap1)	*MATa his3-1,leu2-0, met15-0, ura3-0, TRP1Δ::HIS3, Spt16-9*MYC*	This study
MP024 (Spt16-MYC in *Nap1Δ*)	*Isogenic to MP026 but Nap1 is deleted with KanMX4*	This study

Supplemental Table S2: Regions examined by histone H2B and H3 ChIP.

Primer Set	Chromosomal Location	Primer Set Description	HBRΔ Occupancy	Expression Increase
Control	Chromosome 6<br>53579 – 53878	*ACT1* ORF	Similar to WT	No change
A	Chromosome 10<br>606484 – 606679	*IME1/RPL43B* promoter	Depleted	No change
B	Chromosome 10<br>332839 – 333035	*ZAP1/YJL055W* promoter	Depleted	No change
C	Chromosome 12<br>252289 – 252517	*SPT8* coding region	Depleted	No change
D	Chromosome 13<br>458111 – 458324	*SNO1/SNZ1* promoter	Depleted	*SNO1*, 4.7-fold<br>*SNZ1*, 4.0-fold
E	Chromosome 8<br>296136 – 296317	5' end of *HXT5* coding region	Depleted	7.2-fold

MIQE information for qPCR experiments:

Item to check	Importance	Item to check	Importance
Experimental design		qPCR oligonucleotides	
Definition of experimental and control groups<br>**In this study, we measured the tagged HBR mutant histone against tagged wild type H2B and a control untagged H2B.**	E	Primer sequences<br>PMA1<br>TCAGCTCATCAGCCAACTCAAG<br>CGTCGACACCGTGATTAGATTG<br>SNO1<br>ATAATGACTCAGCCGACAAGG<br>TCGCAGCCAGAATTACAGAC<br>IME1<br>TGCTTACTCACTTTGCGGCT<br>GCGGTAGCAAAGGACAAACG<br>YAR035W<br>TGGAAAACCATCGTCGTCGT<br>CAGGAAATTGTCAGTGCGCC	E
Number within each group<br>**3 or more**	E	RTPrimerDB identification number	D
Assay carried out by the core or investigator's laboratory?<br>**By the investigator's laboratory in the core**	D	Probe sequences	D[4]
Acknowledgment of authors' contributions	D	Location and identity of any modifications<br>**None**	E
Sample		Manufacturer of oligonucleotides<br>**Fisher**	D
Description<br>**Yeast whole cell extracts**	E	Purification method<br>**Desalted**	D
Volume/mass of sample processed<br>**100uL for IP, 10uL for input**	D	qPCR protocol	
Microdissection or macrodissection	E	Complete reaction conditions<br>**ABI 7500 FAST**<br>**Quantitative ΔΔCt**<br>95°→(95°→60° (40x))→95°→60°→95°→60°<br>4' 5s 30s 15s 1' 15s 15s	E
Processing procedure<br>**Following formaldehyde crosslinking, yeast cells were spun down, lysed by bead beating, sonicated, and IP'ed using FLAG antibody. Extracts were proteinase K digested, crosslinking was reversed, and DNA extracted by PCI followed by RNAseA digestion**	E	Reaction volume and amount of cDNA/DNA<br>**ddH_2O 2.05µL**<br>**Eva Green 7.50µL**<br>**10x Rox 0.45µL**<br>**DNA 2µL**	E
If frozen, how and how quickly?<br>**Extracts were frozen at -80° by placing in the freezer**	E	Primer, (probe), Mg^{2+}, and dNTP concentrations<br>**Primer: 0.5mM**<br>**The rest as in**	E

Item to check	Importance	Item to check	Importance
		**commercial Eva Green Mastermix (Biotium)**	
If fixed, with what and how quickly?<br>**1% Formaldehyde for 15 minutes**	E	Polymerase identity and concentration<br>**Cheetah hot-start Taq polymerase**	E
Sample storage conditions and duration (especially for FFPE[2] samples)<br>**Yeast pellets and extracts were stored at -80°**	E	Buffer/kit identity and manufacturer<br>**Fast Eva Green qPCR Master Mix; Biotium**	E
Nucleic acid extraction<br>**PCI**		Exact chemical composition of the buffer	D
Procedure and/or instrumentation<br>**ABI 7500 FAST Quantitative ΔΔCt**<br>95°→(95°→60°(40x))→95°→60°→95°→60°<br><u>4’</u> <u>5s</u> 30s 15s 1’ 15s 15s	E	Additives (SYBR Green I, DMSO, and so forth)<br>**10x ROX**	E
Name of kit and details of any modifications<br>**Fast Eva Green qPCR Master Mix +0.3x Rox; Biotium**	E	Manufacturer of plates/tubes and catalog number<br>**96-well plates from Fisher Scientific Cat# AB-1900**	D
Source of additional reagents used<br>**N/A**	D	Complete thermocycling parameters<br>**ABI 7500 FAST Quantitative ΔΔCt**<br>95°→(95°→60°(40x))→95°→60°→95°→60°<br><u>4’</u> <u>5s</u> 30s 15s 1’ 15s 15s	E
Details of DNase or RNase treatment<br>**Samples were treated with 0.33mg/mL RNAseA at 37° for 15-20 minutes**	E	Reaction setup (manual/robotic)<br>**Manual**	D
Contamination assessment (DNA or RNA)<br>**RNA is not visible following treatment on agarose gel**	E	Manufacturer of qPCR instrument<br>**ABI 7500 fast from Applied Biosystems**	E
Nucleic acid quantification<br>**~10-100ng/uL for IP, ~250-350ng/uL for input**	E	qPCR validation	
Instrument and method<br>**ABI 7500 FAST Quantitative ΔΔCt**<br>95°→95°→60°→95°→60°→95°→60°<br><u>4’</u> <u>5s</u> 30s 15s 1’ 15s 15s	E	Evidence of optimization (from gradients)	D

Item to check	Importance	Item to check	Importance
Purity (A_{260}/A_{280})	D	Specificity (gel, sequence, melt, or digest)<br><br>**Melt Curve**	E
Yield<br>**~10-100ng/uL for IP, ~250-350ng/uL for input**	D	For SYBR Green I, C_q of the NTC<br>**NTC fails to amplify**	E
RNA integrity: method/instrument<br>**N/A**	E	Calibration curves with slope and *y* intercept<br>**Slope:-3.379**<br>**Y intercept: 41.19**	E
RIN/RQI or C_q of 3′ and 5′ transcripts<br>**N/A**	E	PCR efficiency calculated from slope<br>**>97.676%**	E
Electrophoresis traces<br>**RNA is not visible on agarose gel**	D	CIs for PCR efficiency or SE	D
Inhibition testing ($C_q$dilutions, spike, or other)<br>**$C_q$dilutions**	E	r^2 of calibration curve<br>**0.995**	E
Reverse transcription<br>**N/A**		Linear dynamic range<br>**9 logs**	E
Complete reaction conditions	E	C_q variation at LOD<br>**Average SD of C_q for duplicate reactions 0.07 cycles**	E
Amount of RNA and reaction volume	E	CIs throughout range	D
Priming oligonucleotide (if using GSP) and concentration	E	Evidence for LOD<br>**Amplification detected in 100% of input samples**	E
Reverse transcriptase and concentration	E	If multiplex, efficiency and LOD of each assay<br>**N/A**	E
Temperature and time	E	Data analysis	
Manufacturer of reagents and catalogue numbers	D	qPCR analysis program (source, version)<br>**ABI 7500 Software**	E
C_qs with and without reverse transcription	D[3]	Method of $C_q$determination<br>**ΔΔCt**	E
Storage conditions of cDNA	D	Outlier identification and disposition<br>**Data was not excluded**	E
qPCR target information		Results for NTCs<br>**No amplification**	E
Gene symbol<br>**PMA1**<br>**SNO1**<br>**IME1**<br>**YAR035W**	E	Justification of number and choice of reference genes	E
Sequence accession number	E	Description of normalization method	E

Item to check	Importance	Item to check	Importance
		**%IP/Input**	
Location of amplicon<br>**PMA1 chrVII:482495-482618**<br><br>**SNO1 chrXIII:458150+458282**<br><br>**IME1 chrX:606860+606930**<br><br>**YAR035W chrI:191347+191443**	D	Number and concordance of biological replicates<br>**At least 3**	D
Amplicon length<br>**PMA1 124bp**<br>**SNO1 133bp**<br>**IME1 71bp**<br>**YAR035W 97bp**	E	Number and stage (reverse transcription or qPCR) of technical replicates<br>**2 technical replicates on qPCR plate**	E
In silico specificity screen (BLAST, and so on)<br>**BLAST**	E	Repeatability (intraassay variation)<br>**SD =0.02-0.2 cycles**	E
Pseudogenes, retropseudogenes, or other homologs?<br>**No**	D	Reproducibility (interassay variation, CV)	D
Sequence alignment	D	Power analysis	D
Secondary structure analysis of amplicon	D	Statistical methods for results significance<br>**t-test/ANOVA**	E
Location of each primer by exon or intron (if applicable)	E	Software (source, version)<br>**Graphpad Prism 6**	E

SUPPLEMENTARY FIGURES AND LEGENDS

HBR-repressed genes (521) **Random set of 521 genes**

A

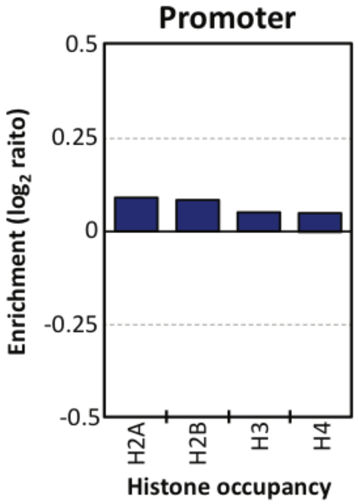

C

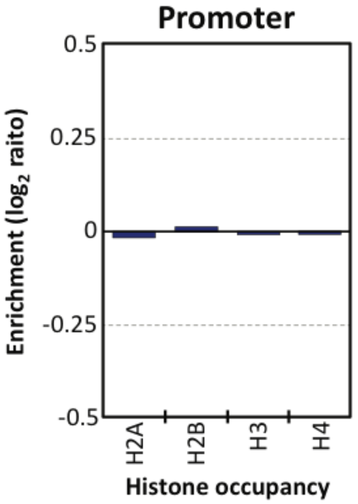

B

Chromatin Enrichment/Depletion			
Promoter			
Histone Modification	Rank Percentile	Trend	P-value
H2A Occupancy	56.6%	**Enriched**	**$<10^{-4}$**
H2B Occupancy	59.6%	**Enriched**	**$<10^{-11}$**
H3 Occupancy	58.6%	**Enriched**	**$<10^{-9}$**
H4 Occupancy	57.7%	**Enriched**	**$<10^{-7}$**

D

Chromatin Enrichment/Depletion			
Promoter			
Histone Modification	Rank Percentile	Trend	P-value
H2A Occupancy	49.8%	None	0.8875
H2B Occupancy	50.1%	None	0.9522
H3 Occupancy	50.0%	None	0.9745
H4 Occupancy	51.1%	None	0.4958

Supplementary Figure S1. HBR-repressed genes have high levels of histone occupancy in their promoters. (**A**) Graph showing average enrichment of histone occupancy in the promoter regions of 521 HBR-repressed genes. Histone occupancy data is from published ChIP-chip experiments stored in the ChromatinDB database (2). (**B**) Statistical analysis using the ChromatinDB indicates that core histone occupancy is significantly enriched in HBR-repressed genes relative to the rest of the genome. (**C** and **D**) No significant enrichment of histone occupancy in the promoters of 521 randomly selected genes. Analysis of histone occupancy was performed using ChromatinDB, as described above.

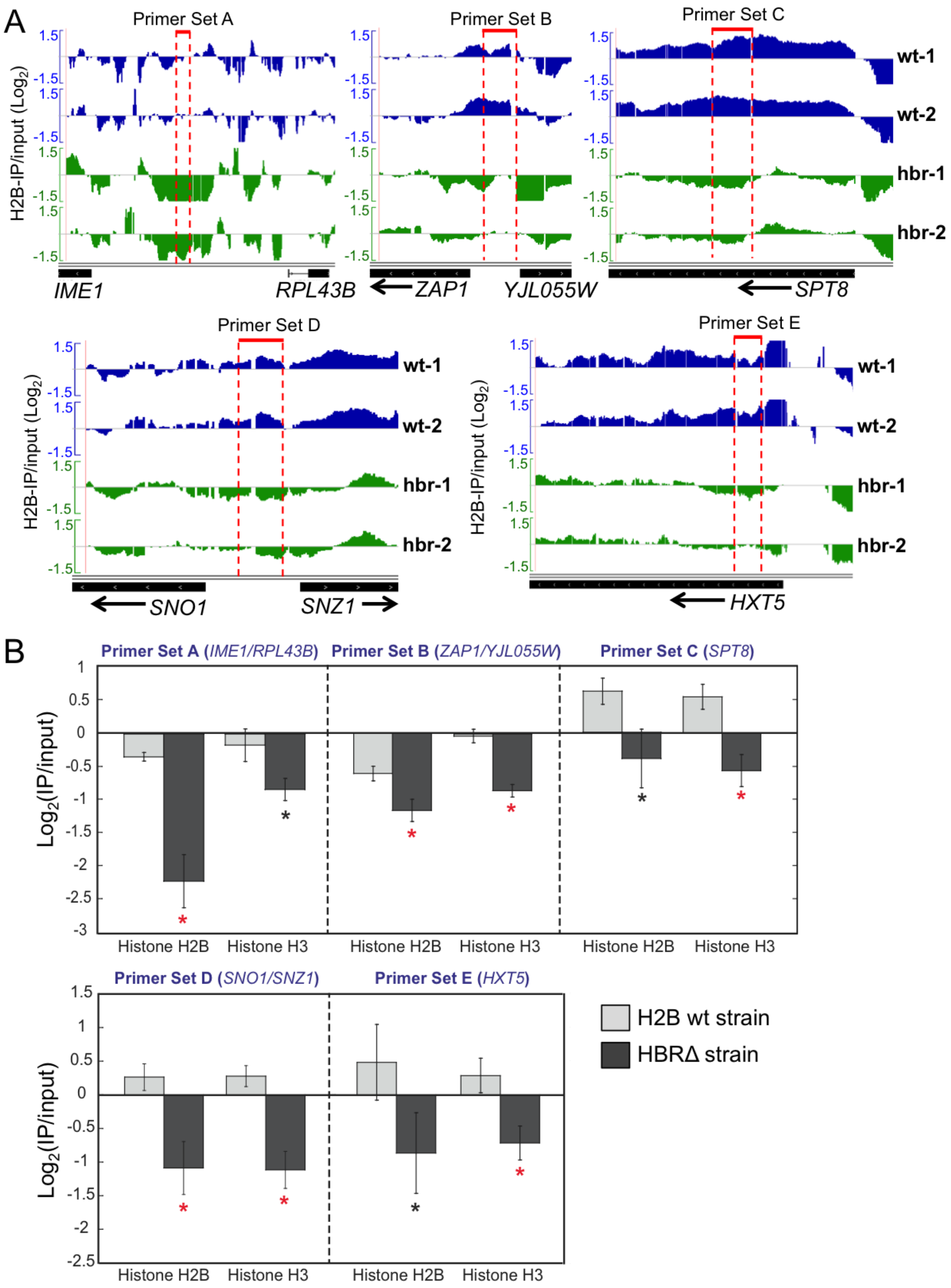

A
Primer Set A
Primer Set B
Primer Set C
H2B-IP/input (Log2)
wt-1
wt-2
hbr-1
hbr-2
IME1
RPL43B
ZAP1
YJL055W
SPT8
Primer Set D
Primer Set E
SNO1
SNZ1
HXT5
B
Primer Set A (IME1/RPL43B)
Primer Set B (ZAP1/YJL055W)
Primer Set C (SPT8)
Log2(IP/input)
Histone H2B
Histone H3
Primer Set D (SNO1/SNZ1)
Primer Set E (HXT5)
H2B wt strain
HBRΔ strain

Supplementary Figure S2. Decreases in H2B occupancy in HBRΔ mutant was confirmed by ChIP-PCR experiments. (**A**) H2B ChIP-chip data for five genomic regions that were depleted in H2B occupancy in the HBRΔ mutant. Wt-1 and wt-2 and hbr-1 and hbr-2 represent two independent wild-type H2B and HBRΔ mutant IP experiments. Data were visualized using IGV (3). (**B**) Analysis of H2B and H3 occupancy using ChIP-PCR. PCR amplified regions are indicated in part **A**. ChIP was performed using either anti-H2B (Histone H2B) or anti-H3 (Histone H3) antibodies. ChIP PCR signal was normalized to an *ACT1* internal control PCR product, as previously described (4). Black asterisks: $P < 0.05$; red asterisks: $P < 0.01$.

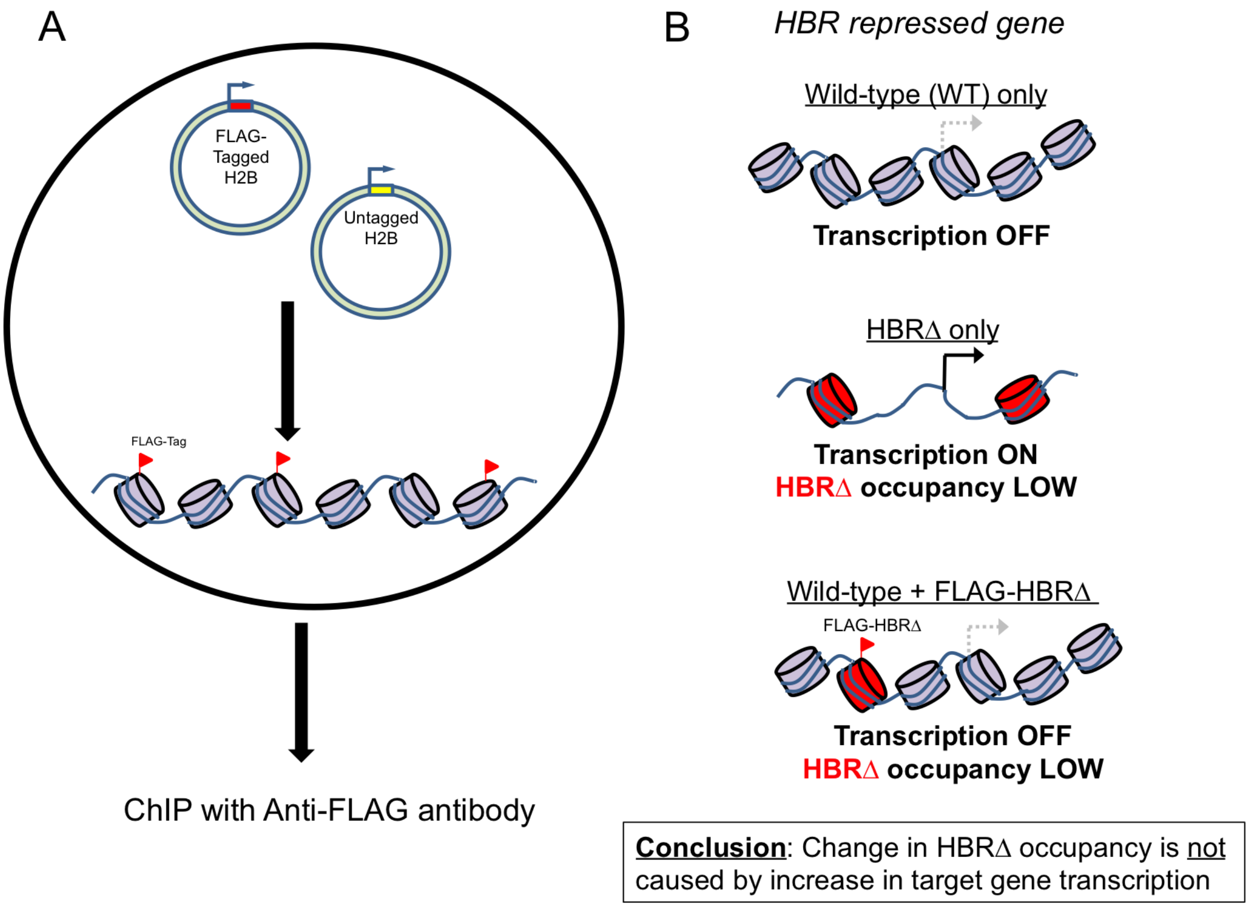

Supplementary Figure S3. (**A**) Schematic showing two-plasmid system for measuring histone occupancy of HBR mutant in a wild-type cell. The HBR mutant is FLAG tagged so it can be specifically detected by ChIP using an anti-FLAG antibody. The presence of the untagged wild-type H2B eliminates the HBR mutant phenotypes that might affect histone occupancy. (**B**) Expected histone occupancy levels for the HBR mutant in the two-plasmid system (i.e., Wild-type + FLAG-HBRΔ) if the decrease in FLAG-HBR occupancy is a direct/intrinsic effect of the HBR mutant, as opposed to a side effect of the increase in transcription of HBR repressed genes.

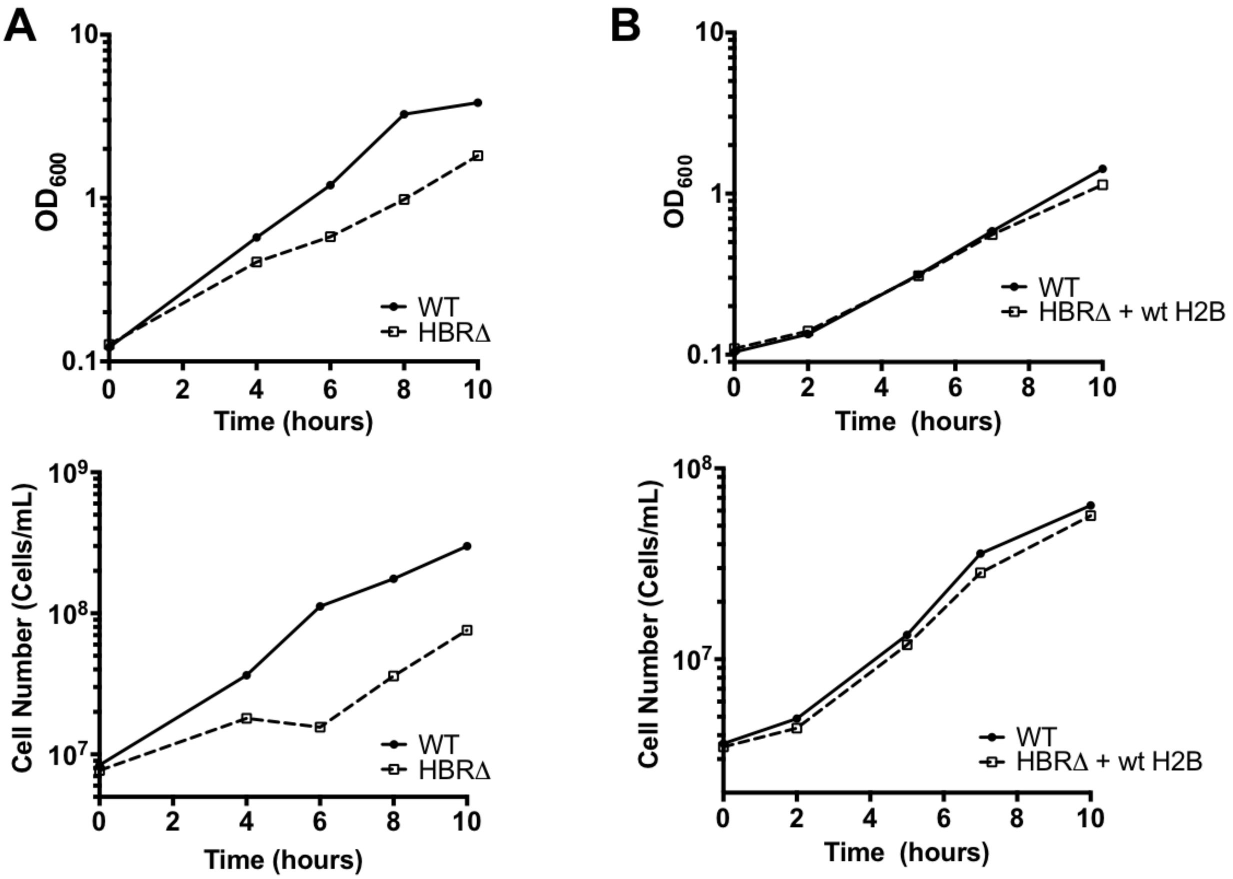

Supplementary Figure S4. Expression of wild-type H2B in the HBR mutant restores growth. (**A**) Growth of WT and HBRΔ mutant strains. Representative growth curves are shown both using OD600 measurements and cell counting. (**B**) Same as **A**, except growth of yeast cells was measured in yeast strains also expressing WT H2B in the 'two-plasmid' system.

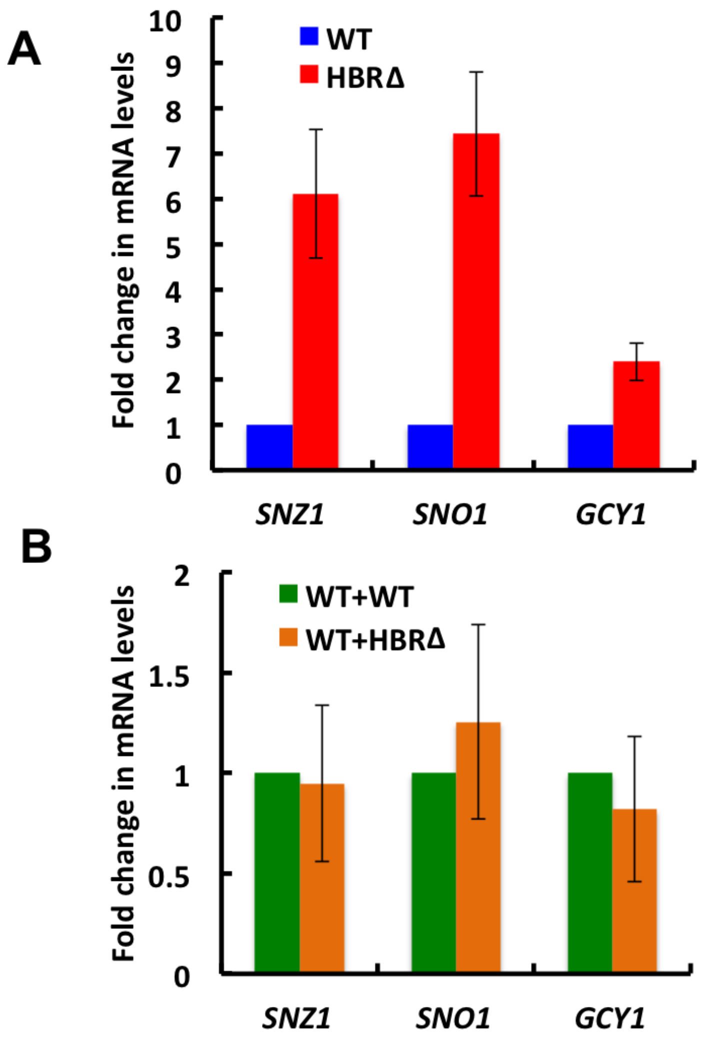

Supplementary Figure S5. Expression of wild-type H2B in the HBR mutant restores transcriptional repression to HBR-repressed genes. (**A**) Fold change in mRNA levels of three HBR-repressed genes (*SNZ1*, *SNO1*, and *GCY1*) in HBRΔ mutant cells relative to wild-type. *ACT1* was used as an internal control. (**B**) Same as **A**, except mRNA levels of HBR repressed genes were measured in yeast strains also expressing WT H2B in the 'two-plasmid' system.

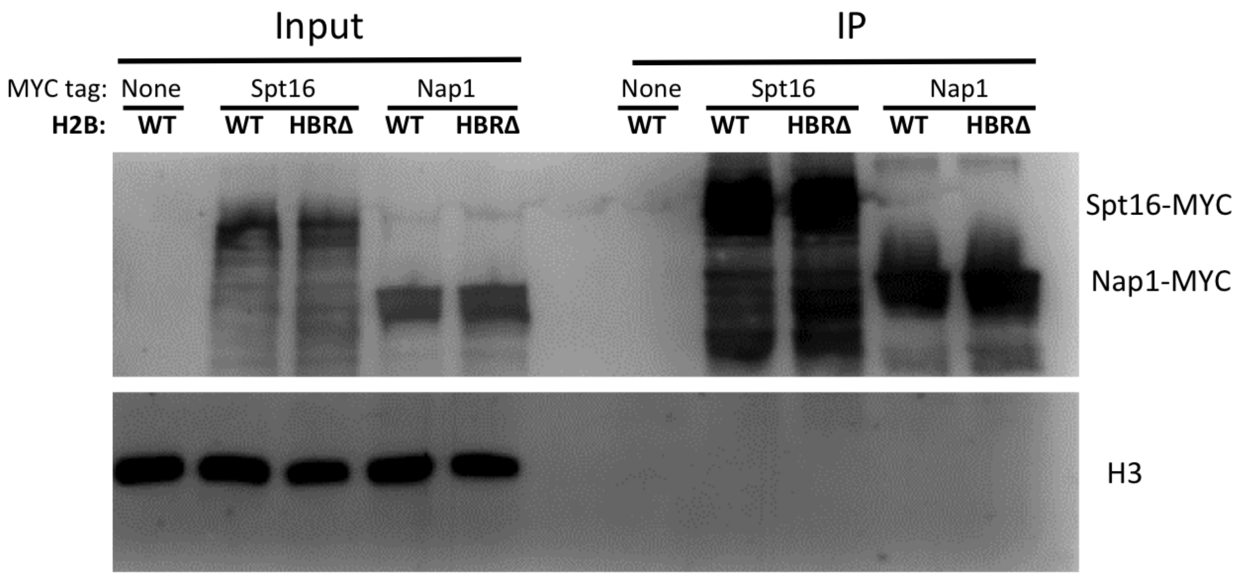

Supplementary Figure S6. Histone H3 is not associated with FACT (Spt16) or Nap1 *in vivo*. Co-IP assay was performed using either wild-type H2B (WT) or HBRΔ (HBR) cell lysates. Spt16 or Nap1 was pulled down with anti-MYC agarose beads.

A

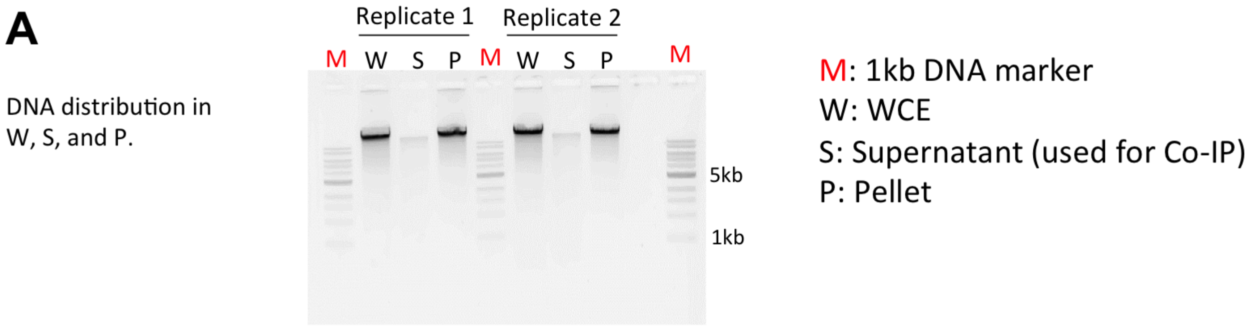

B

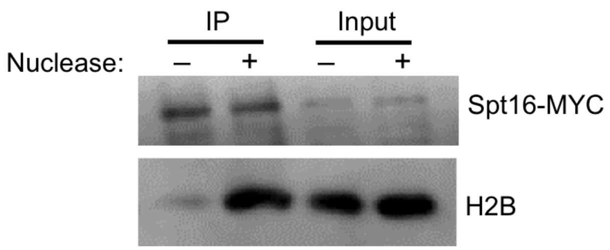

Supplementary Figure S7. Co-IP of histones by FACT is independent of DNA. (**A**) Fractionation of yeast lysates and detection of DNA in each fraction. Yeast whole cell lysate (W) was centrifuged extensively (13,200 rpm, 10') and separated into supernatant (S) and pellet (P). The supernatant fraction is used for Co-IP assays. Yeast DNA was isolated from each fraction and separated on a 1% agarose gel. The 1kb DNA marker (M) was shown on the gel. This experiment was performed twice. (**B**) Co-IP of wild-type H2B bound to FACT (Spt16-Myc) following treatment of the yeast cell lysate with nuclease. Samples in + lanes were treated with 320 U/mL Pierce Universal Nuclease for 30 minutes at 4° C prior to centrifugation and immunoprecipitation.

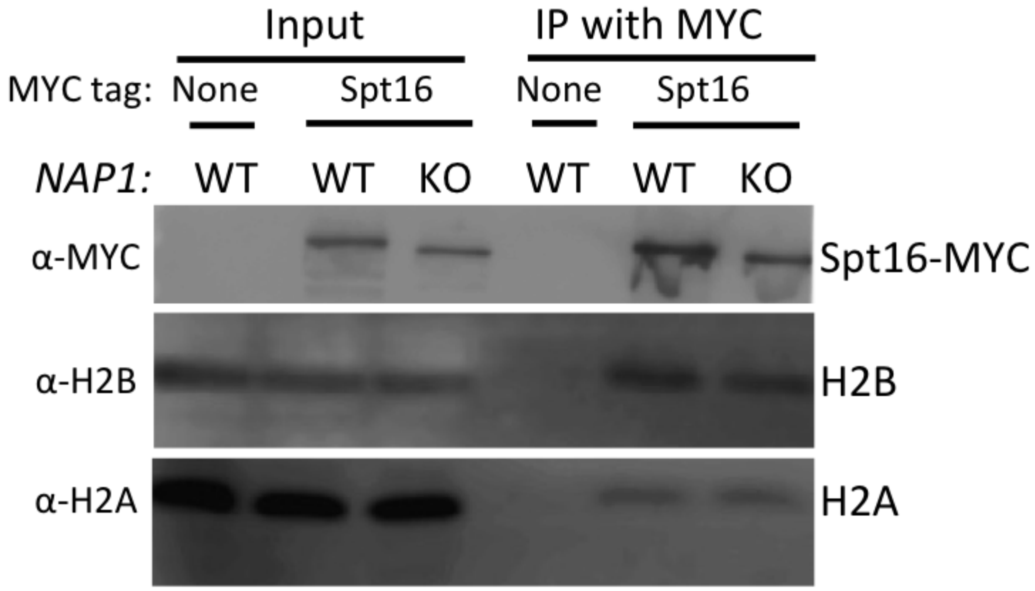

Supplementary Figure S8. Deletion of yeast *Nap1* gene does not improve the binding of Spt16 to H2A/H2B. *NAP1* gene is deleted in the Spt16-MYC strain. Co-IP is performed with anti-MYC antibody to compare FACT-H2A/H2B interaction between wild-type (WT) and *NAP1* knockout (KO).

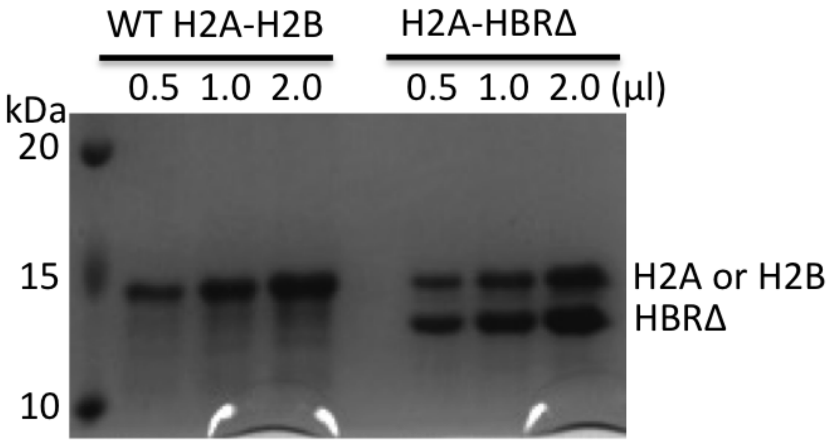

Supplementary Figure S9. Reconstituted H2A-H2B and H2A-HBRΔ histone dimers. The concentrations for WT and mutant dimers are 72 μM and 120 μM, respectively. Proteins are separated on a 15% SDS polyacrylamide gel and stained with Coomassie blue.

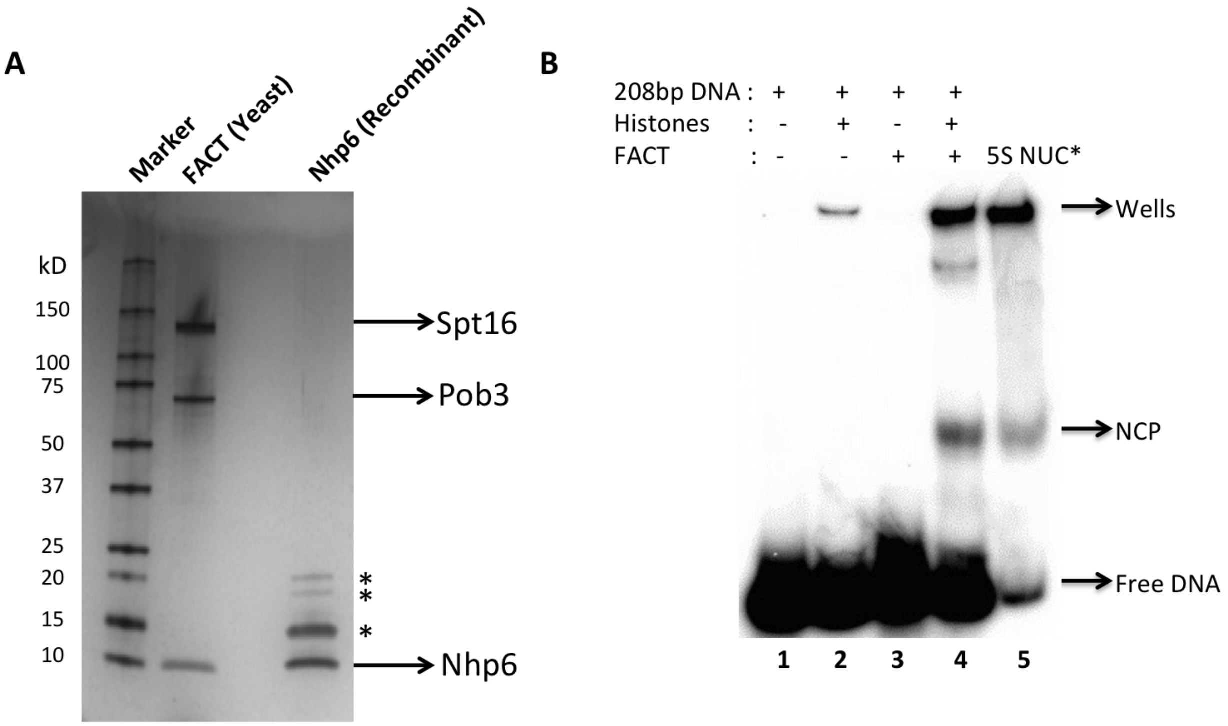

Supplementary Figure S10. Purified yeast FACT is able to assemble nucleosomes *in vitro*. (**A**) Yeast FACT (~200 ng) was resolved on a 4-20% gradient SDS gel. The major components of FACT, Spt16 and Pob3, were detected. A third band was also shown on the gel. Based on its mass and a comparison with recombinant Nhp6 expressed in E. coli, this band appears to be the yeast Nhp6 protein. *Denotes contaminating proteins from purified Nhp6. (**B**) ^{32}P-labeled 5S DNA (50 ng) is incubated with 0.15 pmol wild-type histone octamer alone (lane 2), purified FACT alone (100 ng, lane 3), or both histone octamer and FACT. 'NCP' indicates the position of assembled nucleosomes, and '5S NUC*' indicates nucleosomes formed by standard salt dialysis.

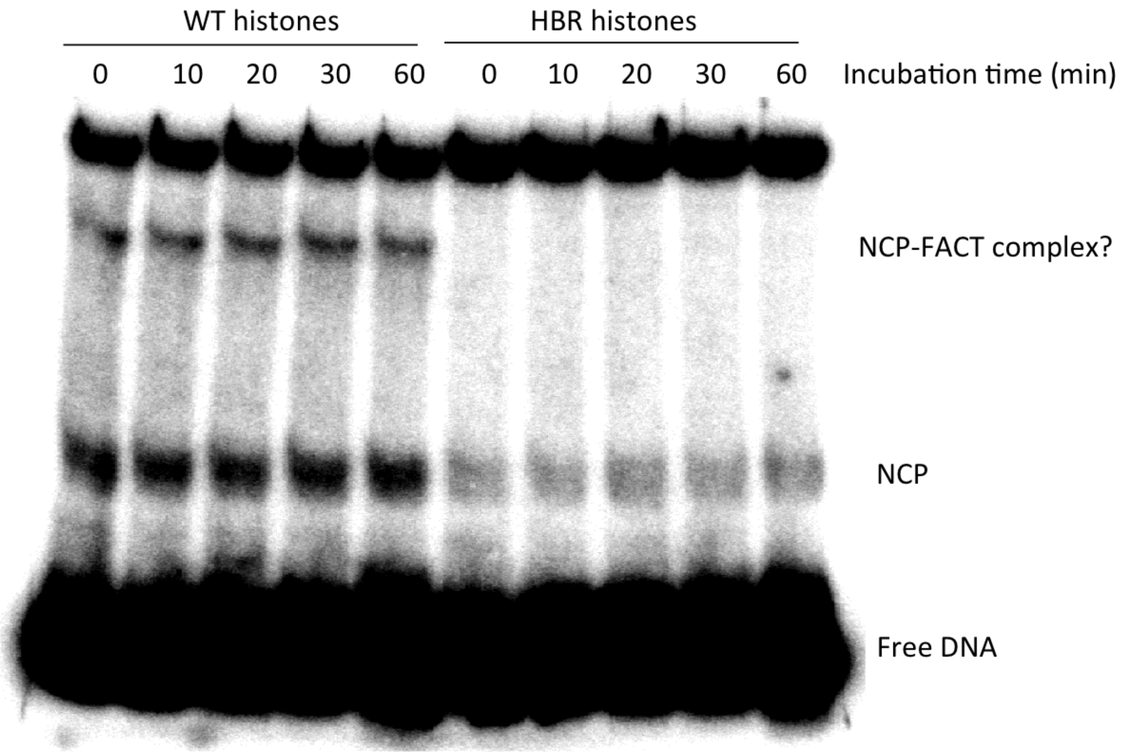

Supplementary Figure S11. Time-course of 5S mono-NCP formation by FACT. Components of the 5S mono-nucleosome assembly reaction are mixed and incubated at 30 ^{0}C. Aliquots are taken at different time points during the incubation. A super-shifted band between wells and the NCP band is seen in WT but not in the mutant. A similar super-shifted band is also seen in other reactions (e.g., Fig. 5A).

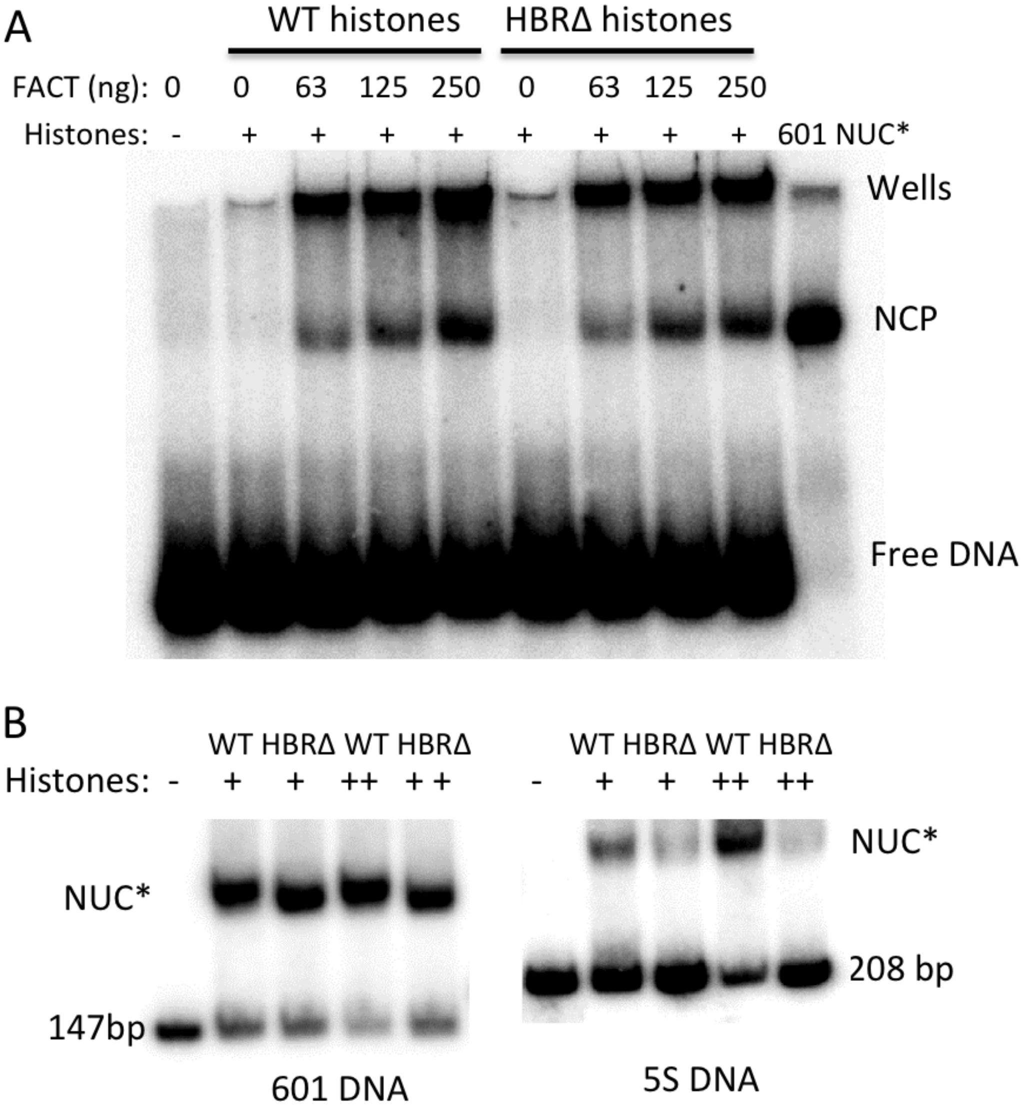

Supplementary Figure S12. The nucleosome assembly defect with HBR-deleted histones can be overcome by using the high affinity Widom-601 DNA. (**A**) 601 mono-nucleosome assembly by yeast FACT with wild-type (WT) or mutant (HBRΔ) histone octamers. Histones are incubated with ^{32}P-labeled 147 bp 601 DNA (40 ng) in the presence of different concentrations of yeast FACT. After incubation, nucleosomes are separated from free DNA on an 8% native polyacrylamide gel. '601 NUC*' indicates 601 nucleosomes assembled via salt dialysis. (**B**) Nucleosome formed by salt-dialysis on 147 bp 601 (left) or 208 bp 5S (right) DNA. For each DNA substrate, wild-type (WT) and mutant (HBRΔ) histone octamers are compared side by side. '+' indicates the molar ratio between histone and DNA is roughly 1.0 : 1.0, and '++' indicates the ratio is 1.2 : 1.0.

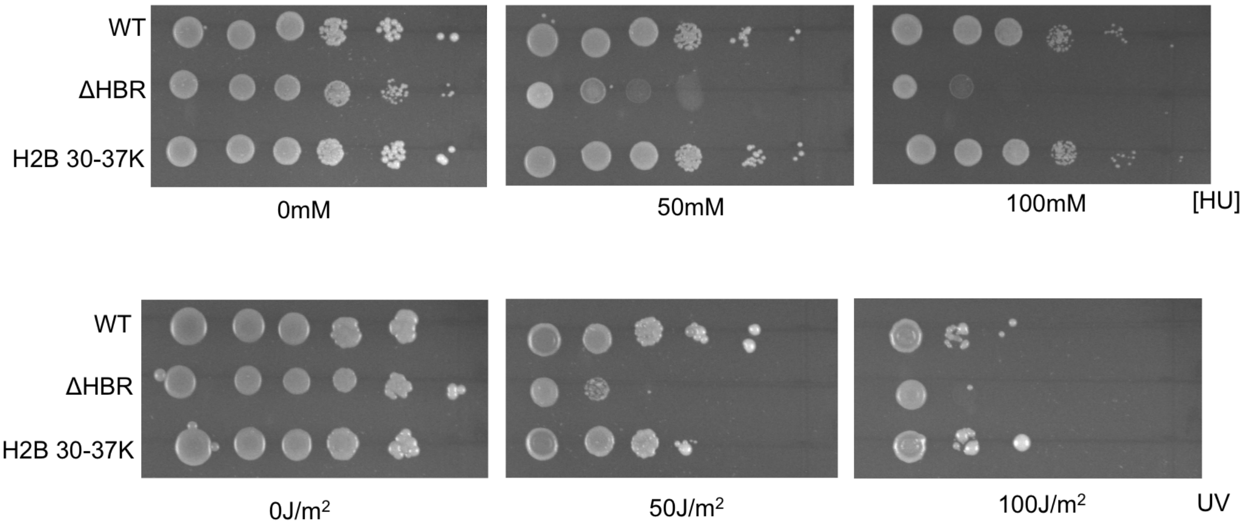

Supplementary Figure S13. HBR domain deletion, but not substitution with lysine, causes UV and hydroxyurea (HU) sensitivity phenotypes. Ten-fold serial dilutions of yeast containing wild type H2B (WT), H2B Δ30-37 (HBRΔ), or H2B 30-37 lys were spotted onto SC+Ade plates (**A**) containing the indicated concentrations of HU or (**B**) exposed to the indicated dose of UV-C light. Images were taken after 2-3 days at 30°C.

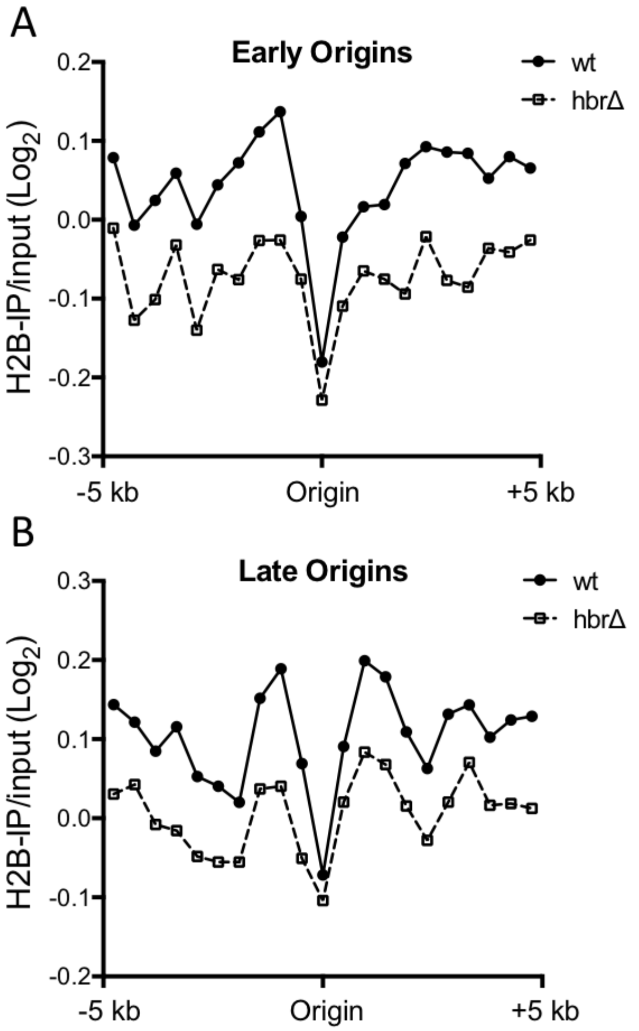

Supplementary Figure S14. HBR domain affects histone occupancy adjacent to early and late replication origins. (**A**) Plot showing average H2B occupancy profile adjacent to early replicating origins in wt and HBRΔ mutant strains. Data represent the average ChIP-chip probe data for 500 bp bins flanking early replication origins. (**B**) Same as part **A**, except late replicating origins were analyzed. Replication origin coordinates were from (5).

Supplementary References

1. Parra, M.A., Kerr, D., Fahy, D., Pouchnik, D.J. and Wyrick, J.J. (2006) Deciphering the roles of the histone H2B N-terminal domain in genome-wide transcription. *Molecular and cellular biology*, **26**, 3842-3852.
2. O'Connor, T.R. and Wyrick, J.J. (2007) ChromatinDB: a database of genome-wide histone modification patterns for Saccharomyces cerevisiae. *Bioinformatics*, **23**, 1828-1830.
3. Robinson, J.T., Thorvaldsdottir, H., Winckler, W., Guttman, M., Lander, E.S., Getz, G. and Mesirov, J.P. (2011) Integrative genomics viewer. *Nature biotechnology*, **29**, 24-26.
4. Kyriss, M.N., Jin, Y., Gallegos, I.J., Sanford, J.A. and Wyrick, J.J. (2010) Novel functional residues in the core domain of histone H2B regulate yeast gene expression and silencing and affect the response to DNA damage. *Molecular and cellular biology*, **30**, 3503-3518.
5. Soriano, I., Morafraile, E.C., Vazquez, E., Antequera, F. and Segurado, M. (2014) Different nucleosomal architectures at early and late replicating origins in Saccharomyces cerevisiae. *BMC genomics*, **15**, 791.
